# Distinct and Overlapping Neuroprotective Efficacy of Silk Lutein and Sericin-Derived Oligopeptides from Yellow Silk Cocoons in Rodent Models of A*β*-Induced and Age-Related Cognitive Decline

**DOI:** 10.3390/ijms27072986

**Published:** 2026-03-25

**Authors:** Pornnarin Taepavarapruk, Virakboth Prum, Manote Sutheerawattananonda

**Affiliations:** 1 Department of Physiology, Faculty of Medical Science, Naresuan University, Phitsanulok 65000, Thailand; pornnarint@nu.ac.th; 2 School of Food Technology, Institute of Agricultural Technology, Suranaree University of Technology, Nakhon Ratchasima 30000, Thailand; prumvirakboth9@gmail.com

**Keywords:** cognitive decline, sericin-derived oligopeptides, silk lutein, brain aging, synaptic plasticity, multi-target silk bioactives

## Abstract

Yellow silk cocoons of Bombyx mori provide two distinct bioactive classes: the carotenoid silk lutein (SL) and sericin-derived oligopeptides (SDOs). Their comparative efficacy and mechanisms in promoting cognitive health remain uncharacterized. This study compared the neuroprotective and cognitive-enhancing effects of SL and SDOs through chronic oral administration in two rodent models: an amyloid-beta (A*β*_25–35_)-induced amnesia model in mice and a natural aging model in rats. Cognitive function was assessed using the Morris Water Maze (MWM) and Novel Object Recognition (NOR) tests, and underlying mechanisms were investigated via in vivo hippocampal long-term potentiation (LTP) and immunohistochemical analysis for apoptosis markers. Both SL and SDOs significantly ameliorated *Aβ*-induced deficits in recognition and spatial memory. Both substances enhanced spatial memory and LTP in old male rats in the natural aging paradigm, with efficacy comparable to that of donepezil (Don). This LTP-enhancing effect was sex-specific, being prominent in males but absent in aged females, although both sexes showed improved recognition memory. Critically, cognitive impairments in the *Aβ* model were not associated with significant neuronal apoptosis, and the protective effects appeared independent of anti-apoptotic pathways. In conclusion, SL and SDOs are potent cognitive-enhancing agents that mitigate memory deficits in acute neurotoxicity and chronic aging models. Their primary mechanism appears to be a robust enhancement of hippocampal synaptic plasticity rather than apoptosis prevention, positioning them as powerful synaptoprotective agents. These findings validate the potential to upcycle this agro-industrial byproduct into high-value nutraceuticals for promoting healthy brain aging.

## 1. Introduction

The intertwined epidemics of age-related cognitive decline, including its most devastating form, Alzheimer’s disease (AD), and metabolic syndrome represent a profound global health challenge of the 21st century. With the world’s population aging at an unprecedented rate, the number of individuals living with dementia is projected to triple by 2050, posing an immense socioeconomic and emotional burden on societies worldwide [1,2]. Despite decades of research, the pharmaceutical pipeline has yielded disappointing outcomes, with current therapeutic options offering only modest symptomatic relief without altering the course of the underlying neurodegenerative cascade [3,4,5]. While conventional anti-dementia drugs, such as acetylcholinesterase inhibitors such as donepezil, rivastigmine, and galantamine [6], provide symptomatic benefits, they are often hindered by significant limitations. These include high costs, limited global availability, and a range of adverse effects, such as gastrointestinal distress, insomnia, and bradycardia [7,8], that may be poorly tolerated in elderly patients with pre-existing cardiovascular conditions [9]. Furthermore, these agents typically target single pathways and fail to address the multifactorial nature of brain aging [10]. Therapeutic challenges have prompted a strategic turn in biomedical research toward preventative and multi-target approaches, with an emphasis on identifying novel bioactive compounds from natural sources that can enhance the brain’s resilience against age-related effects [11,12]. Cognitive decline arises from two interconnected drivers: overwhelming oxidative stress and the A*β* neurotoxic cascade [13,14,15]. The brain’s metabolic demands and lipid-rich environment heighten its vulnerability to age-dependent ROS damage, causing direct neuronal dysfunction [16]. A*β* aggregation impairs synaptic plasticity and triggers neuroinflammatory pathways, ultimately resulting in the widespread neuronal loss characteristic of AD. In addition, cognitive decline is also driven by a progression from functional synaptic failure to structural neuronal loss [17]. In the early stages of neurodegeneration, soluble A*β* oligomers interfere with hippocampal long-term potentiation (LTP) and synaptic remodeling, leading to impaired memory encoding and retrieval before overt physical damage occurs [18]. The synergy between chronic neuroinflammation and oxidative stress triggers apoptotic pathways, resulting in the irreversible loss of neurons in key cognitive centers. Understanding these distinct phases is crucial for developing interventions that can preserve synaptic integrity before irreversible damage ensues. Consequently, animal models employing intracerebral A*β* administration have become a cornerstone for preclinical screening, effectively replicating key aspects of cognitive impairment and allowing for the evaluation of potential neuroprotective agents [19,20]. This critical need for novel therapeutic avenues forms the basis of our broader research program, which is dedicated to exploring the neuroprotective potential of underutilized, sustainable bioactives. At the forefront of this exploration is the yellow silk cocoon of *Bombyx mori*, an agro-industrial byproduct traditionally discarded during silk production in Thailand [21,22]. Our research idea is founded on the hypothesis that this single, sustainable source contains at least two chemically distinct classes of compounds with potential neuroprotective properties: protein-based SDOs and the carotenoid SL. The upcycling of this byproduct into high-value nutraceuticals represents a significant opportunity for both sustainable innovation and public health, especially the region that yellow silk rearing is a common practice.

Our previous work has begun to uncover the systemic benefits of these compounds. We have demonstrated that SDOs, low-molecular-weight peptides derived from sericin, possess potent antioxidant and antihypertensive activities in both normotensive and L-NAME-induced hypertensive rat models. Notably, these antihypertensive actions are mechanistically linked to potent vasorelaxant effects, mediated through the enhancement of the eNOS/NO/sGC pathway and modulation of calcium channels [21,23,24]. Furthermore, SDOs have been shown to exhibit significant hypoglycemic effects in streptozotocin (STZ)-induced diabetic rats [22]. Mechanistically, these dual benefits are strongly associated with their ability to inhibit both ACE and dipeptidyl peptidase IV (DPP-IV) [25]. Crucially, recent studies have demonstrated the remarkable stability of these peptides under various storage conditions, with both their physical properties and dual enzyme-inhibitory activities remaining robust over six months even at elevated temperatures, underscoring their viability as a functional food ingredient [26]. This vascular benefit is highly pertinent, as maintaining robust cerebral blood flow and endothelial health is fundamental to preventing age-related cognitive impairment. Given that hypertension is a major modifiable risk factor for dementia [27,28], these findings provide a strong, albeit indirect, rationale for their potential role in cognitive health. Although indirect, these vascular effects may contribute to improved cerebral perfusion and resilience, potentially supporting cognitive maintenance. In parallel, the same cocoon yields SL, a unique protein-bound form of a lipophilic carotenoid [29]. Lutein is known to accumulate in the brain from an early age to adulthood and exert neuroprotective effects, partly by mitigating A*β*-induced oxidative stress and apoptosis in neuronal cell lines [30,31,32]. Crucially, the protein-conjugated structure of SL has been shown to confer superior stability and antioxidant capacity compared to free lutein from conventional plant sources [33,34], suggesting its potential as a robust neuroprotective agent. According to a recent study, SL has antihypertensive properties in aging rats [35]. However, its comparative efficacy against SDOs in in vivo models of cognitive decline has never been investigated.

Although preliminary findings and the existing literature suggest the neuroprotective potential of these compounds individually, their comparative efficacy and the extent to which they utilize distinct or overlapping molecular mechanisms remain to be elucidated. SDOs (peptides) and SL (a carotenoid) are fundamentally different molecules. Understanding their relative strengths, for instance, in restoring memory, enhancing synaptic plasticity, or preventing neuronal death, is essential for guiding future development, whether as standalone agents or as a synergistic combination. Therefore, the primary objective of this study was to perform a comparison of the neuroprotective and cognitive-enhancing efficacies of SL and SDOs, derived from the same sustainable source, across both acute neurotoxic (A*β*_25–35_-induced) and chronic physiological (natural aging) models of cognitive decline. Through the evaluation of multiple cognitive domains and the underlying mechanisms of hippocampal synaptic plasticity and apoptosis, this research provides a critical mechanistic rationale for developing these compounds into novel, evidence-based nutraceuticals aimed at mitigating the profound challenge of age-related cognitive decline. This study provided the first direct comparative analysis of whether these agro-industrial byproducts can provide multi-target cognitive-enhancing strategies. By exploring these bioactives together, this study establishes a definitive framework for utilizing yellow silk cocoons as a comprehensive source for brain nutraceuticals.

## 2. Results and Discussion

### 2.1. Comparative Radical Scavenging Potency, Reducing Power, and Metal-Chelating Capacity of SDOs and SL

The in vitro antioxidant characterization of yellow silk bioactives showed a distinct and complementary profile between SL and SDOs. In terms of direct radical scavenging and reducing power, SL demonstrated exceptional potency, with $IC_{50}$ values in the microgram range for both ABTS (324.15±10.8 μg/mL) and FRAP (30.33±0.06 μg/mL) assays (Table 1). This superior potency of SL is closely linked to its exceptionally high Total Phenolic Content (TPC) of 147.67±0.90 mg GAE/g [33]. The presence of such a high phenolic concentration suggests that SL functions as a carotenoid-polyphenol complex, where the phenolic constituents work synergistically with the lutein core to enhance radical neutralization [36]. In contrast, SDOs exhibited these activities with 2100.00±0.07 μg/mL and 9070.00±0.07 μg/mL, respectively, indicating that SL is approximately 300 to 1000 times more potent by weight as a direct electron donor and radical scavenger [37]. However, a critical functional divergence was observed in the metal-chelating assay, where SDOs showed significant activity ($IC_{50}=22,850.00\pm 0.07$ μg/mL), while no activity was detected for SL.

This lack of chelating capacity in SL is attributed to its chemical structure as a xanthophyll carotenoid: its terminal rings possess only hydroxyl groups, which lack the necessary charge and spatial orientation to form stable coordinate bonds with transition metal ions in an aqueous environment [38]. Furthermore, the high lipophilicity of SL limits its interaction with metal ions in water-based assay systems. In contrast, the chelating success of SDOs is driven by their peptide nature, specifically the presence of carboxyl-rich amino acids such as aspartic and glutamic acid, which act as effective ligands to sequester metals.

From a neuroprotective perspective, this mechanistic dichotomy is highly advantageous for addressing the multifactorial nature of brain aging. The lipophilic nature and high potency of SL position it as a primary defense against lipid peroxidation within neuronal membranes [39]. Simultaneously, the metal-chelating properties of SDOs provide a preventative mechanism against metal-induced neurotoxicity, which is known to catalyze A*β* aggregation and the generation of highly reactive hydroxyl radicals via the Fenton reaction [40,41]. Together, these findings provide a strong biochemical rationale for the development of a dual-action silk-derived bioactive complex that targets both the lipid and protein-based oxidative damage characteristic of the aging brain.

### 2.2. General Physiological Observations

**Figure 1:**
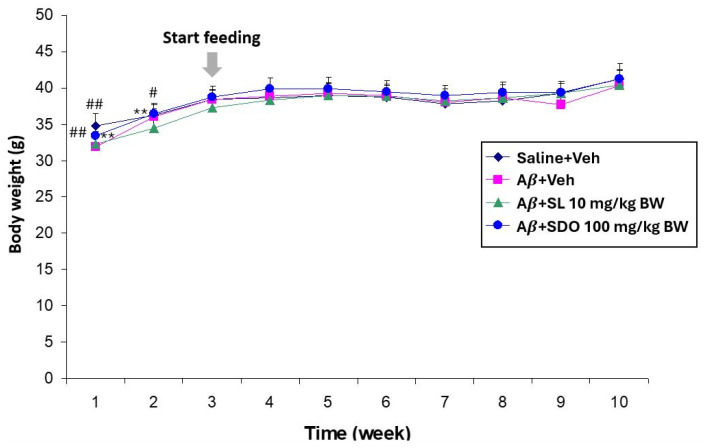
Effect of chronic administration of SL and SDOs on body weight in the A*β*_25–35_-induced amnesia model. Body weights of ICR mice were monitored weekly for 8 weeks following intracerebroventricular (i.c.v.) surgery. Data are presented as mean ± SEM (n = 12 per group). Statistical significance was determined by one-way ANOVA followed by Dunnett’s multiple comparison test. ** *p* < 0.01 compared to Saline + Veh group; # *p* < 0.05, ## *p* < 0.01 compared to A*β* + Veh group.

To ascertain the general safety and tolerability of the test compounds, body weights were monitored throughout the duration of all experimental models. In the A*β*_25–35_-induced amnesia model, long-term oral administration of either SL (10 mg/kg BW) or SDOs (100 mg/kg BW) for 8 weeks shows some significant differences in body weight at weeks 1 and 2 of Saline + Veh and A*β* + SDO groups compared to the control and A*β* + Veh control groups (Figure 1). However, after week 2, all groups exhibited a normal growth trajectory, suggesting that the treatments did not adversely affect general health or appetite.

Similarly, in the 3-month natural aging study, chronic administration of SL (10 mg/kg BW), SDOs (100 mg/kg BW), or the positive control, Donepezil (1 mg/kg BW), did not induce significant weight loss in either aged male or female rats when compared to their respective aged vehicle-treated controls (Table 2). While aged male rats showed a slight, non-significant decrease in body weight across all groups, consistent with typical age-related changes, aged female rats exhibited a slight increase in body weight, which was statistically significant for the SL, SDO, and Donepezil groups compared to their initial weights but not significantly different from the aged vehicle control group. These data collectively indicate that long-term administration of both SL and SDOs at the tested doses is well-tolerated and does not produce systemic toxicity in either young adult ICR mice or aged rats.

A prerequisite for any potential nutraceutical is its long-term safety. Our findings consistently demonstrated that chronic oral administration of both SL and SDOs, for up to 3 months in aged rats, was well-tolerated. The absence of significant adverse effects on body weight or general health across all experimental paradigms is a crucial first step, suggesting that these compounds do not induce systemic toxicity or alter fundamental metabolic processes at the effective doses. This favorable safety profile is consistent with previous toxicological studies on SL and SDOs [21,35] and the established safety of dietary lutein [42], providing a solid foundation for their potential use as long-term dietary supplements for cognitive health.

### 2.3. Comparative Protective Effects of SL and SDOs Against Aβ_25–35_-Induced Cognitive Impairment in Mice

#### 2.3.1. SL and SDOs Prevent A*β*-Induced Deficits in Recognition Memory

**Figure 2:**
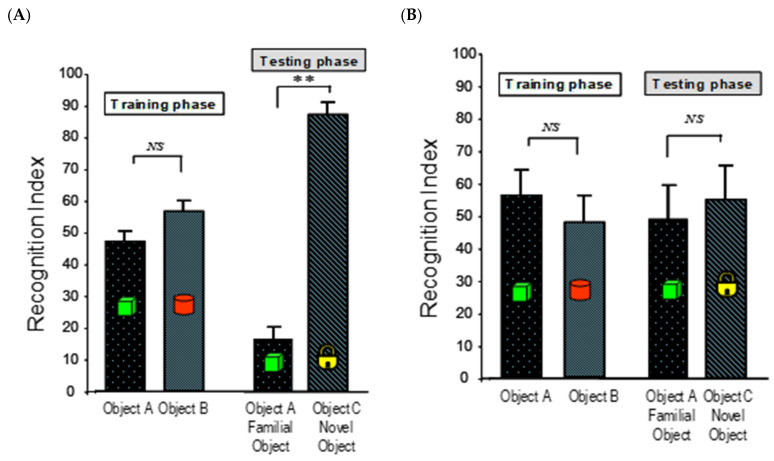

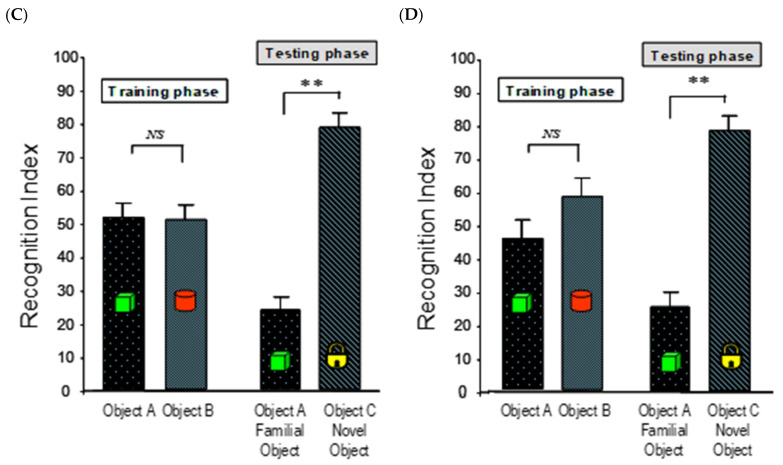
Protective effects of SL and SDOs on recognition memory in the A*β*_25–35_-induced amnesia model: (**A**) Saline + Veh, (**B**) A*β* + Veh, (**C**) A*β* + SL 10 mg/kg BW, (**D**) A*β* + SDO 100 mg/kg BW. The bar chart displays the RI from the NOR test performed after 8 weeks of treatment and subsequent i.c.v. surgery. Data are presented as mean ± SEM (n = 12 per group). Statistical significance was determined using a paired sample *t*-test. ** *p* < 0.01 compared to object A within each group and phase. “NS” indicates no significant difference.

The NOR test was employed to assess the protective effects of the compounds on non-spatial recognition memory, relying on the animal’s innate preference for novelty. Improvements in recognition index (RI) reflect enhanced information processing and retrieval [43]. As expected, the Saline + Veh group demonstrated robust memory, spending significantly more time exploring the novel object compared to the familiar one (Figure 2A). In contrast, i.c.v. injection of A*β*_25–35_ resulted in a profound memory deficit in the A*β* + Vehicle (Veh) group, as evidenced by their inability to discriminate between the novel and familiar objects (RI) (not significantly different from 50%) (Figure 2B), confirming the successful induction of amnesia.

Chronic pre-treatment with either SL (10 mg/kg BW) or SDOs (100 mg/kg BW) for 8 weeks completely prevented this A*β*-induced memory impairment. Mice in both the A*β* + SL 10 mg/kg BW and A*β* + SDO 100 mg/kg BW groups showed a strong and significant preference for the novel object, with RIs of 76.91 ± 4.12% and 75.64 ± 4.27%, respectively (Figure 2C,D). These values were significantly higher than the chance-level performance of the A*β* + Vehicle group and were statistically indistinguishable from the performance of the healthy control group, indicating a complete preservation of recognition memory function.

A key finding of this study is the protective efficacy of both SL and SDOs in the A*β*_25–35_-induced amnesia model. The intracerebroventricular injection of A*β* is a well-established model that mimics the synaptic dysfunction and memory impairment seen in the early stages of Alzheimer’s disease [44]. Our results clearly show that mice pre-treated with either SL or SDOs were completely protected from the deficits in recognition memory (NOR), performing at a level indistinguishable from healthy controls. This protective effect likely stems from the antioxidant properties inherent to both compounds. A*β* peptides are known to induce a state of intense localized oxidative stress by promoting the generation of reactive oxygen species (ROS), which in turn leads to lipid peroxidation, protein oxidation, and ultimately, synaptic failure [45,46]. Both lutein, a known carotenoid antioxidant that crosses the blood–brain barrier [32], and SDOs, which have demonstrated significant radical scavenging activity (Table 1), likely counteract this initial oxidative insult, thereby preserving synaptic integrity and function in the face of A*β* pathology.

#### 2.3.2. SL and SDOs Ameliorate A*β*-Induced Impairment in Spatial Learning and Memory

**Figure 3:**
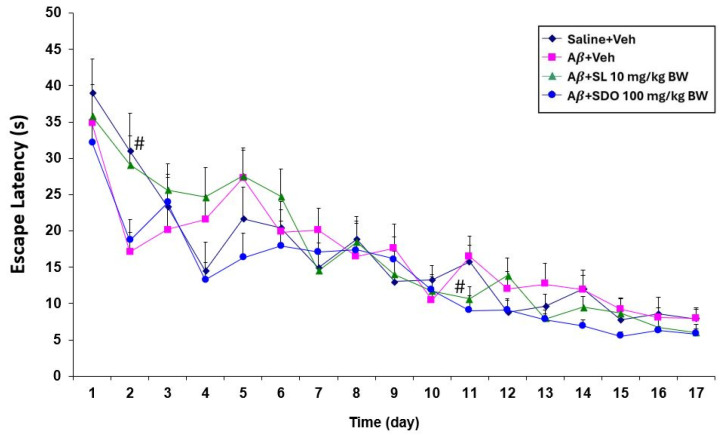
Comparison of mean escape latencies across experimental groups during the training phase. Data are presented as mean ± SEM (n = 12). Statistical significance was determined by one-way ANOVA followed by Dunnett’s multiple comparison test. # *p* < 0.05 compared to A*β* + Veh group.

The MWM test was performed to evaluate the effects on hippocampal-dependent spatial learning and memory over a 17-day training period (Figure 3). The reduction in escape latency during training and an increased search time in the target quadrant during probe trials indicate superior spatial navigation and memory retention [43]. All groups, including the control and the $A\beta_{25-35}$-treated groups (A*β* + Veh, A*β* + SL 10 mg/kg BW, and A*β* + SDO 100 mg/kg BW), exhibited similar mean escape latencies when released from three different starting positions. A consistent reduction in escape latency was observed over time across all animal groups, suggesting that baseline learning capacity was uniform among the groups before the induction of cognitive impairment.

**Figure 4:**
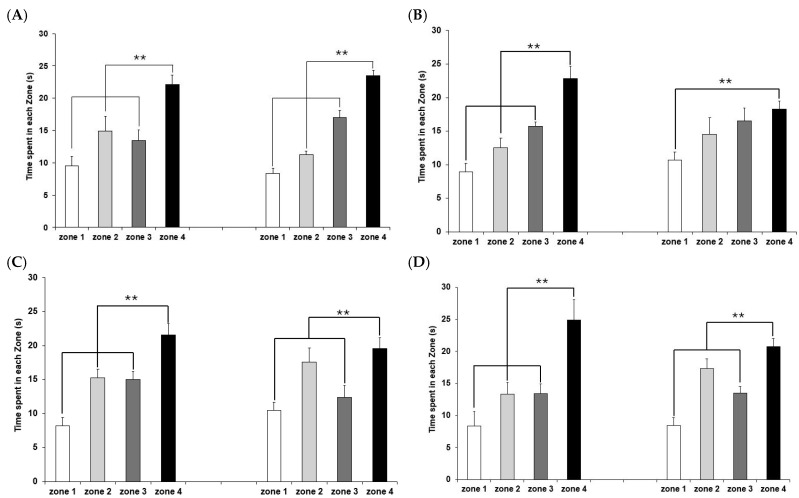
Spatial memory performance during the probe trial test, represented by the percentage of time spent in each zone for (**A**) Saline + Veh, (**B**) A*β* + Vehicle, (**C**) A*β* + SL 10 mg/kg BW, and (**D**) A*β* + SDO 100 mg/kg BW. Comparisons are shown between pre-injection (**left**) and post-injection (**right**) phases. Zone 4 indicates the target quadrant. Data are presented as mean ± SEM (n = 12). Statistical significance was determined using a paired sample *t*-test. ** *p* < 0.01 compared to time spent in zone 4 within each group and phase.

To assess spatial memory retention, a probe trial was performed on day 18 by removing the platform. The mice were allowed to swim freely for 90 s, and the time spent in each zone was quantified as a percentage of the total duration to determine the animals’ ability to retrieve spatial information. The A*β* + Veh group showed impaired spatial memory, spending about similar amount of time in all four quadrants (Figure 4B). In contrast, the control group spent significantly more time in the target quadrant where the platform was previously located (Figure 4A). Both the A*β* + SL 10 mg/kg BW and A*β* + SDO 100 mg/kg BW groups demonstrated a significant improvement in memory retention compared to the A*β* + Veh group, indicating that both treatments effectively protected against A*β*-induced spatial memory deficits (Figure 4C,D).

#### 2.3.3. Assessment of Apoptosis in the A*β*-Induced Model

**Figure 5:**
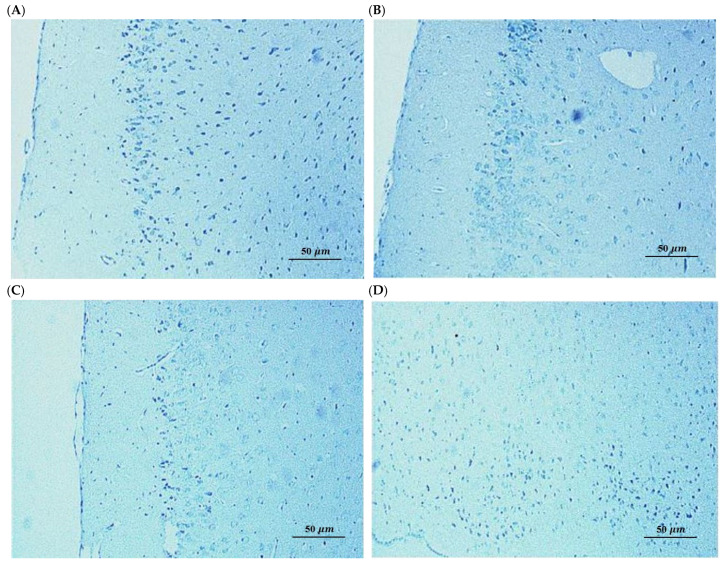
Absence of significant neuronal apoptosis in the hippocampus of A*β*_25–35_-injected mice. Representative photomicrographs of the hippocampal CA1 region stained with FragELTM DNA fragmentation detection kit (TUNEL assay): (**A**) Saline + Veh, (**B**) A*β* + Veh, (**C**) A*β* + SL, and (**D**) A*β* + SDO groups. No significant increase in TUNEL-positive cells (indicative of apoptosis) or overt signs of neuronal loss and pyknotic nuclei were observed in the A*β* + Veh group compared to the control group. Scale bar = 50 µm.

To determine whether the observed cognitive deficits in the A*β*_25–35_ model were associated with neuronal cell death and whether the protective effects of SL and SDOs involved anti-apoptotic mechanisms, we performed immunohistochemical and histological analysis of hippocampal sections. TUNEL staining provides a highly sensitive assessment for detecting DNA fragmentation, which is a hallmark of apoptosis [47]. In preclinical models of cognitive decline, this method is essential to determine whether memory deficits are driven by irreversible structural loss or by functional synaptic dysfunction. Microscopic examination of brain sections stained with the FragELTM DNA fragmentation detection kit (Sigma-Aldrich, Darmstadt, Germany), a method for detecting apoptotic cells (TUNEL assay), revealed a negligible presence of TUNEL-positive cells across all experimental groups, including the A*β* + Veh group (Figure 5B). Qualitative morphological assessment confirmed that hippocampal neurons remained structurally intact, with no overt signs of cell shrinkage or pyknotic nuclei observed in A*β*-injected mice compared to the Saline + Veh group. This observation is consistent with the findings of Wang et al. (2019) [48], who demonstrated that intrahippocampal injection of A*β* oligomers can induce significant cognitive impairment in the absence of detectable neuronal loss.

**Figure 6:**
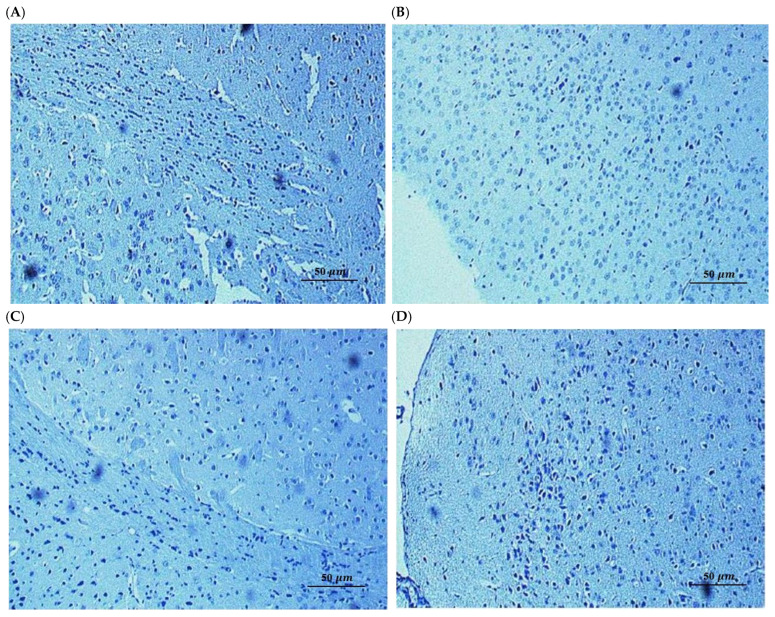
Representative photomicrographs of the hippocampal CA1 region stained with Hematoxylin & Eosin. Scale bar = 50 µm. (**A**) Saline + Veh, (**B**) A*β* + Veh, (**C**) A*β* + SL, and (**D**) A*β* + SDO groups. No significant increases in signs of neuronal loss and pyknotic nuclei were observed in the A*β* + Veh group compared to the control group. Scale bar = 50 µm.

This finding was further corroborated by Hematoxylin and Eosin staining (Figure 6), which assesses general neuronal morphology. H&E staining is the standard protocol for assessing general tissue architecture and identifying overt signs of neurodegeneration, such as cell shrinkage or pyknotic nuclei [49]. The neuronal morphology in the CA1 regions of A*β*-injected mice appeared largely intact and was indistinguishable from that of the Saline + Veh group. We did not observe any overt signs of neurodegeneration, such as widespread neuronal loss, cell shrinkage, or pyknotic nuclei, which are characteristic hallmarks of apoptosis. Representative hippocampal sections showing preserved neuronal morphology and absence of DNA fragmentation, confirming that cognitive deficits were independent of overt neurodegeneration [50].

**Figure 7:**
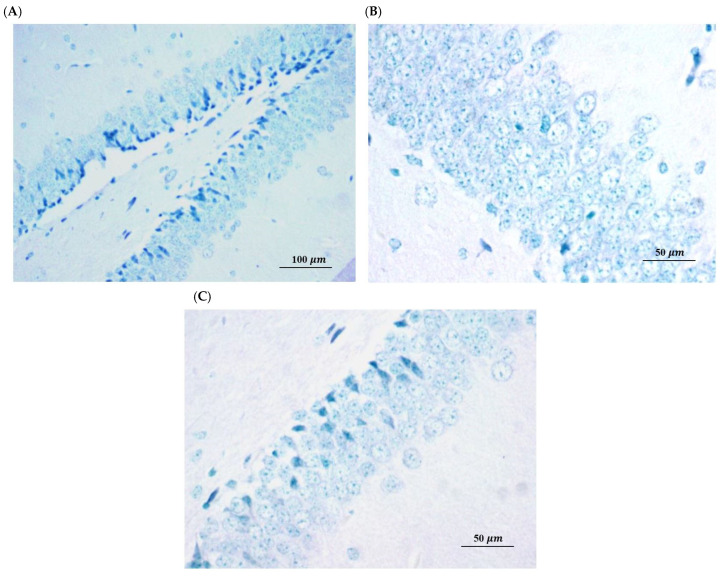
Representative immunohistochemistry (IHC) overview of the hippocampal regions: (**A**) Dentate Gyrus (200×), (**B**) CA1, and (**C**) CA3 (400×) for the A*β* + Veh group to demonstrate the areas being assessed for potential neuronal damage.

To establish whether the observed effects were due to structural preservation or functional enhancement, we performed immunohistochemical analysis for apoptotic markers [51]. This provides a basis for distinguishing between agents that prevent irreversible neuronal loss and those that act by restoring biochemical and synaptic functions in surviving neurons. Immunohistochemical analysis of the hippocampal sub-regions (Dentate Gyrus, CA1, and CA3) in the A*β*-induced group revealed no significant expression of apoptotic proteins, with neuronal layers appearing structurally intact and organized (Figure 7). These findings, consistent with the negative TUNEL results, indicate that the observed cognitive impairments in this model are not a consequence of overt neuronal loss or programmed cell death [52]. Instead, the data suggests that A*β*_25–35_ primarily induces synaptic and biochemical dysfunction rather than neurodegeneration at this stage, implying that the neuroprotective effects of SL and SDOs are likely mediated through the preservation of synaptic integrity and function rather than a direct anti-apoptotic mechanism. The absence of significant apoptosis was consistent across all treatment groups, including those receiving SL and SDOs.

Collectively, these results strongly suggest that the profound cognitive impairments induced by a single i.c.v. injection of A*β*_25–35_ in this model are primarily attributable to synaptic dysfunction rather than overt neuronal loss. Consequently, the robust protective effects of both SL and SDOs are likely mediated through the modulation of synaptic health and function, rather than through the prevention of apoptotic cell death.

Furthermore, our investigation into apoptosis sheds light on the primary mode of A*β*-induced damage in our model. We found no significant evidence of widespread neuronal apoptosis in the hippocampus of A*β*_25–35_-injected mice. This crucial negative result strongly suggests that the cognitive impairments observed are primarily a consequence of synaptic dysfunction rather than overt neuronal loss [53]. This aligns with the modern understanding of Alzheimer’s disease, where synaptic failure is now considered a very early event that precedes widespread cell death by years or even decades [13,54,55]. Consequently, the efficacy of SL and SDOs in this model is consistent with preservation of synaptic function rather than a purely anti-apoptotic one. This positions them as ideal candidates for early intervention, aimed at enhancing synaptic resilience before irreversible neuronal loss occurs.

### 2.4. Therapeutic Effects of SL and SDOs on Age-Related Cognitive Decline in Aged Rats

#### 2.4.1. SL and SDOs Improve Spatial Learning and Memory

**Figure 8:**
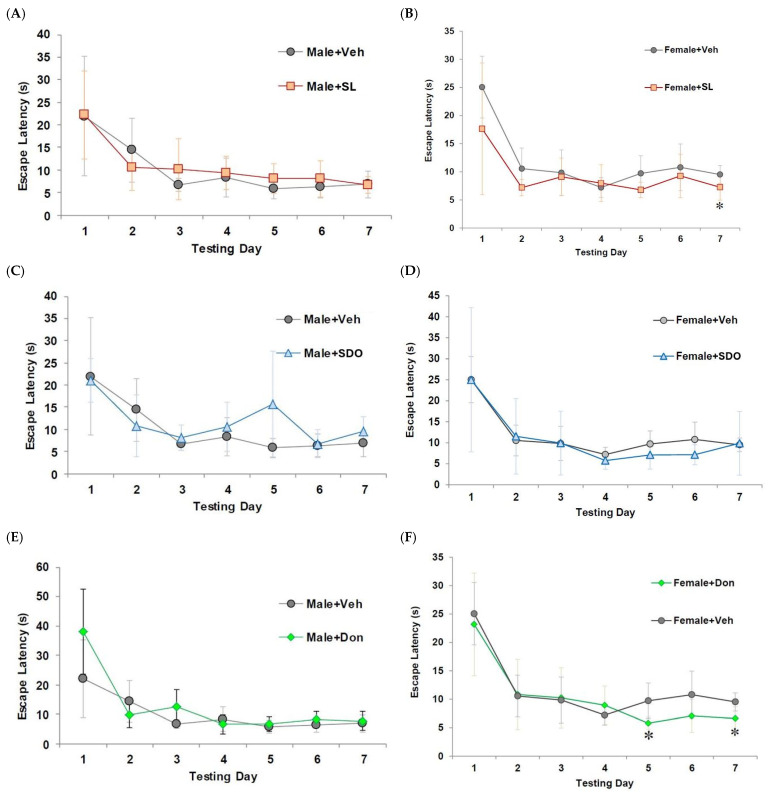
Therapeutic effects of SL, SDOs, and Don on the escape latency: (**A**) Male + SL, (**B**) Female + SL, (**C**) Male + SDO, (**D**) Female + SDO, (**E**) Male + Don, and (**F**) Female + Don, during a 1-week MWM learning trial. Data represents the mean escape latency for each group; all cohorts in both sexes showed similar spatial learning and memory performance by day 7. Data are presented as mean ± SEM (n = 6–8). Statistical significance was determined by using independent-samples *t*-test. * *p* < 0.05 compared to the respective aged Veh group.

To investigate the therapeutic potential of SL and SDOs on pre-existing age-related cognitive deficits, we utilized a natural aging model. In the MWM test, aged male control rats displayed significant impairment in spatial learning, characterized by a flat learning curve and long escape latencies throughout the training week (Figure 8A,C,E). In female rats, chronic 3-month treatment with SL (10 mg/kg BW) and Don (1 mg/kg BW) resulted in a significant improvement in spatial learning. The escape latencies for both treatment groups were progressively shorter and were significantly lower than the Female + Veh group by the final day of training (Figure 8B,D,F).

**Figure 9:**
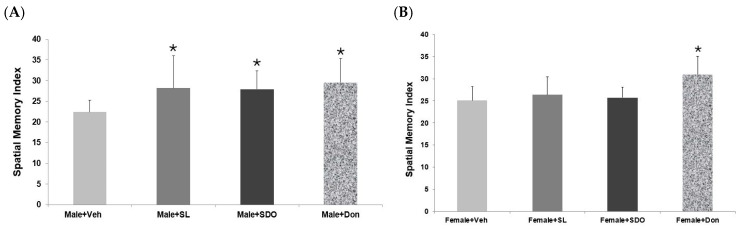
Comparison of spatial memory indices in aged (**A**) male and (**B**) female rats during the MWM probe trial. Data are presented as mean ± SD. Statistical significance was determined by using independent-samples *t*-test. * *p* < 0.05 compared to the Vehicle control group.

In the probe trial, which assesses spatial memory retention, both SL- and SDO-treated aged male rats spent significantly more time in the target quadrant compared to the Male + Veh, demonstrating enhanced spatial memory (Figure 9A). This improvement was statistically comparable to that observed in the Don-treated positive control group, which indicates a potent therapeutic effect on hippocampal-dependent memory in aging males. An interesting sex-specific effect was observed in the MWM test. Unlike in males, the therapeutic effects of SL and SDO on spatial learning and memory in aged female rats were less pronounced and did not reach statistical significance compared to the Female + Veh group (Figure 9B). Only the Don-treated group showed a significant improvement in spatial memory in females.

The most compelling evidence for the therapeutic potential of these compounds may come from the natural aging model. We demonstrated that a 3-month administration of both SL and SDOs significantly improved spatial memory in aged male rats, an effect comparable to the positive control drug, Don. This finding suggests that these compounds may not only protect against an acute insult but also reverse or ameliorate pre-existing, age-related cognitive decline. Interestingly, we observed a pronounced sex-specific effect: the cognitive enhancement in spatial memory was more pronounced in male than in female aged rats. This sexual dimorphism is a well-documented phenomenon in neuroscience and may be linked to the complex interplay between aging, sex hormones, and neuroplasticity [56]. The age-related decline in estrogen, a potent neuroprotective hormone, may render the female brain less responsive to certain therapeutic interventions [57,58].

#### 2.4.2. Sex-Specific Effects on Recognition Memory

**Figure 10:**
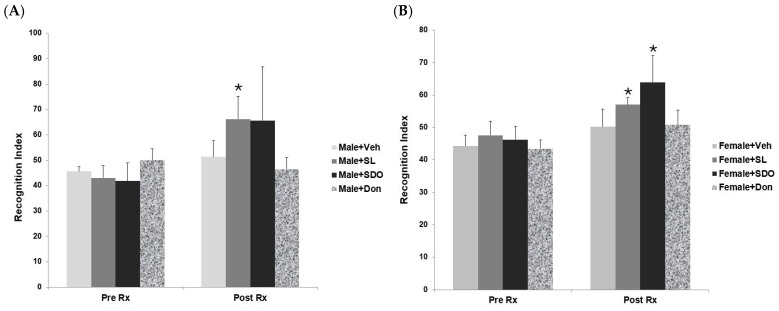
Comparison of object recognition indices in aged (**A**) male and (**B**) female rats during Pre-Rx and Post-Rx phases. The bar chart displays the RI from NOR test before and after 3 months of treatment. Data are presented as mean ± SEM (n = 6–8 per group). Statistical significance was determined using a paired sample *t*-test. * *p* < 0.05 compared to the Pre-Rx value within the same group.

Unlike in the spatial memory test, both sexes showed significant improvement in recognition memory, suggesting that SL and SDOs may modulate different cognitive domains through distinct pathways, some of which are less dependent on hormonal status [59,60]. When assessing recognition memory using the NOR test, both SL and SDOs demonstrated significant efficacy in both sexes. Before treatment (Pre-Rx), all aged groups showed impaired recognition memory. After 3 months of treatment (Post-Rx), both aged male and female rats treated with SL or SDOs exhibited a significantly higher Recognition Index compared to their pre-treatment baseline and also compared to their respective aged vehicle controls (Figure 10A,B). This indicates that while the beneficial effects on complex spatial memory may be sex-dependent, both SL and SDOs are broadly effective at improving the ability to recognize and remember novel objects in both aging male and female brains.

#### 2.4.3. Effects on General Locomotor Activity and Exploratory Behavior

**Figure 11:**
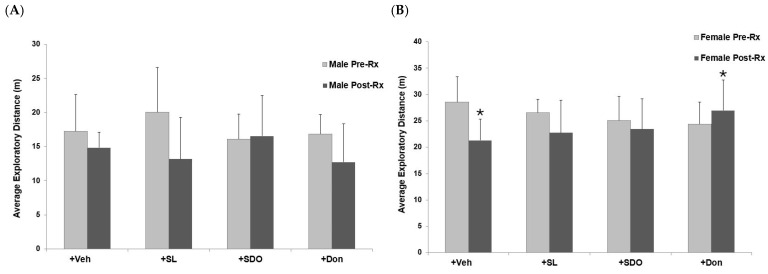
Exploratory behavior and physical performance of aged (**A**) male and (**B**) female rats following administration of SL and SDO extracts from yellow silk cocoons, or Donepezil, assessed by the open-field test. Data are presented as mean ± SEM (n = 6–8 per group). Statistical significance was determined using a paired sample *t*-test. * *p* < 0.05 compared to Pre-Rx value within the same group.

The open-field test was employed to evaluate general locomotor activity and exploratory drive [61]. The effects of yellow silk cocoon extracts and Don on the locomotor activity of aged male and female rats in the open-field test are summarized in Figure 11A,B, respectively. In the Male + Veh, the baseline mean walking distance was 17.3±5.3 m. After three months of Veh administration, the distance decreased to 14.8±2.3 m, which was not statistically significant. Similar results were observed in the Male + SL group, where the exploratory distance changed from 20.1±6.5 m at baseline to 13.2±6.1 m after three months of treatment; however, this decrease did not reach statistical significance. For the Male + SDO group, the mean distance was 16.1±3.7 m before treatment and 16.6±5.9 m after three months, showing no significant difference. Likewise, the Male + Don group exhibited a slight, non-significant reduction in mean distance from 16.9±2.8 m at baseline to 12.7±5.6 m after the three-month period.

In the Female + Veh group, the baseline exploratory distance was 28.6±4.8 m. After three months of vehicle administration, this group exhibited a reduction in average walking distance to 21.3±4.0 m. For the Female + SL group, the exploratory distance decreased significantly from 26.5±2.6 m to 22.7±6.1 m after three months of treatment, suggesting the sign of aging. Similarly, the Female + SDO group showed no significant difference in exploratory performance, with mean distances of 25.1±4.5 m and 23.5±5.7 m before and after treatment, respectively. Moreover, the Female + Don group showed a significant improvement in physical performance, with average exploratory distance increasing from 24.4±4.2 m at baseline to 27.0±5.8 m after three months of administration.

#### 2.4.4. Effects on Motor Coordination and Balance

**Figure 12:**
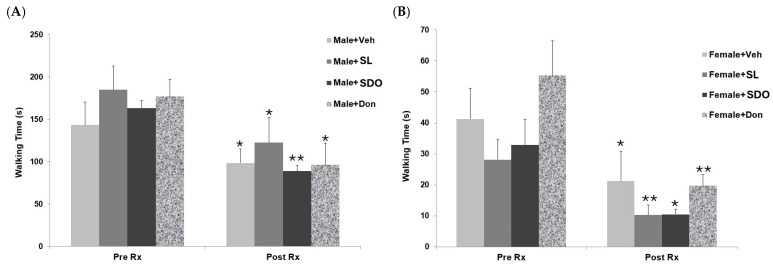
Motor coordination and physical performance of aged (**A**) male and (**B**) female rats assessed by the narrow beam walking test. Data compares the effects of Veh, SL, SDOs, and Don at baseline (Pre-Rx) and following 3 months of treatment (Post-Rx). Data are presented as mean ± SEM (n = 6–8 per group). Statistical significance was determined using a paired sample *t*-test. * *p* < 0.05, ** *p* < 0.01 compared to the Pre-Rx value within the same group.

The beam walking test was utilized as a sensitive measure of fine motor coordination and balance [62]. This allowed us to determine whether the neuroprotective effects of the bioactives extend to the motor system, which is characteristically impaired during natural aging [63]. In the aged Male + Veh, the baseline time required to cross the narrow beam walking 143.1±27.1 s (Figure 12A). After three months of vehicle administration, this group showed a significant improvement in walking speed, with the crossing time decreasing to 98.3±16.5 s. Similar trends were observed across all other experimental groups. The Male + SL group exhibited a significant reduction in crossing time, from 184.8±28.0 s at baseline to 122.4±29.7 s after the three-month treatment period. Notably, the Male + SDO group showed a marked and significant improvement, with the average time decreasing from 163.2±9.1 s to 88.9±7.0 s. Finally, the Male + Don group also demonstrated a significant enhancement in performance, as the crossing time was reduced from 176.9±20.1 s at baseline to 96.2±25.5 s after three months of administration.

Regarding the results in aged female rats, the Female + Veh exhibited a baseline crossing time of 41.3±9.9 s (Figure 12B). Following three months of vehicle administration, this group showed a significant improvement in walking speed, with the time reduced to 21.3±9.4 s. Consistent with these findings, all other experimental groups demonstrated significant enhancements in motor performance. The Female + SL group showed a significant reduction in crossing time, from 28.1±6.5 s at baseline to 10.3±3.2 s after the three-month treatment period. Similarly, the Female + SDO group exhibited a significant decrease in time from 33.0±8.2 s to 10.5±1.8 s. Finally, the Female + Don group showed a marked and significant improvement, with the average crossing time decreasing from 55.4±11.1 s at baseline to 19.8±3.5 s after three months of administration.

#### 2.4.5. Effects on Forelimb Muscle Strength

**Figure 13:**
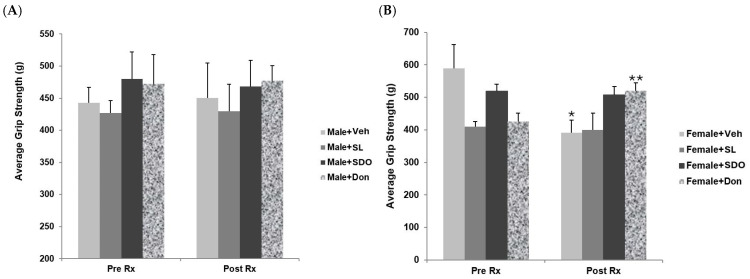
Forelimb grip strength of aged (**A**) male and (**B**) female rats before (Pre-Rx) and after 3 months (Post-Rx) of treatment with vehicle, SL, SDOs, or Don. Data are presented as mean ± SEM (n = 6–8 per group). Statistical significance was determined using a paired sample *t*-test. * *p* < 0.05, ** *p* < 0.01 compared to the Pre-Rx value within the same group.

Grip strength test was performed to assess neuromuscular function and forelimb muscle strength, parameters that worsen during natural aging, serving as an essential indicator of overall physical robustness [64]. In the aged Male + Veh, the baseline forelimb grip strength was 442.6±24.3 g (Figure 13A). After three months of vehicle administration, although a slight increase in grip strength was observed (450.7±53.8 g), the change was not statistically significant. Similarly, the Male + SL group showed a baseline grip strength of 427.2±19.0 g, which marginally increased to 430.0±41.8 g after three months of treatment, showing no significant difference. For the Male + SDO group, the grip strength was 480.3±41.8 g at baseline and 468.2±40.5 g after three months of administration; no significant difference was observed compared to the pre-treatment phase. Finally, the Male + Don group exhibited a baseline value of 472.7±45.4 g and a post-treatment value of 477.1±23.5 g after three months, which also showed no significant difference compared to the initial baseline.

The results for the aged female groups are summarized in Figure 13B. In the Female + Veh group, the baseline grip strength was 589.5±72.1 g. After three months of vehicle administration, a significant decrease to 390.6±38.8 g was observed. In contrast, the Female + SL group showed no significant difference in grip strength, with values of 410.0±15.7 g at baseline and 400.2±50.8 g after three months of treatment. Similarly, the Female + SDO group exhibited no significant change, with grip strength measuring 519.1±20.4 g before treatment and 406.2±42.9 g post-treatment. However, these results differed significantly from the Female + Don group, which demonstrated a significant increase in grip strength from 424.8±26.5 g at baseline to 519.5±25.1 g after three months of administration. Based on these findings, it can be concluded that yellow silk cocoon extracts may not effectively enhance forelimb muscle strength in aged rats of either sex.

#### 2.4.6. Effects of SL and SDOs on Hippocampal Synaptic Plasticity in Aged Rats

To understand the cellular mechanisms underlying these cognitive improvements, we investigated hippocampal LTP, a fundamental form of synaptic plasticity believed to be the cellular basis for learning and memory [65,66,67]. In vivo hippocampal LTP provides the fundamental electrophysiological basis for evaluating SL and SDO efficacy at the cellular level. As the primary mechanism underlying memory formation and synaptic plasticity, the magnitude of LTP induction serves as physiological evidence of the silk bioactives ability to improve the brain’s health. In aged male rats, where cognitive improvements were most pronounced, both SL and SDO treatments robustly restored the impaired LTP to levels seen in young, healthy animals. This provides a powerful mechanistic link, directly connecting the molecular action of the compounds to enhanced synaptic function and, consequently, improved behavioral performance. The restoration of LTP in the chronically aged brain suggests that SDOs can directly target and rejuvenate the endogenous mechanisms of synaptic plasticity that have declined progressively over time.

The robust restoration of LTP observed in this study can be attributed to the complementary physicochemical profiles of SL and SDOs. As established in our previous biochemical characterization, SL exists in a unique protein-bound form that confers superior oxidative stability compared to conventional lutein [33]. Its lipophilic nature allows it to integrate into neuronal membranes, where it directly neutralizes lipid peroxidation, a critical event in A*β*-induced neurotoxicity. Conversely, SDOs operate as a cytosolic and systemic defense. Our previous work demonstrated that these peptides are highly stable against gastrointestinal and plasma hydrolysis [25]. This may allow them to reach the brain in bioactive form. Being hydrophilic, SDOs neutralize ROS within the aqueous cytosolic environment. By stabilizing the redox environment across both the membrane (SL) and the cytosol (SDOs), these bioactives protect the functional integrity of the synapse, providing a cohesive multi-target strategy to mitigate cognitive impairment and complex neurodegenerative diseases.

**Figure 14:**
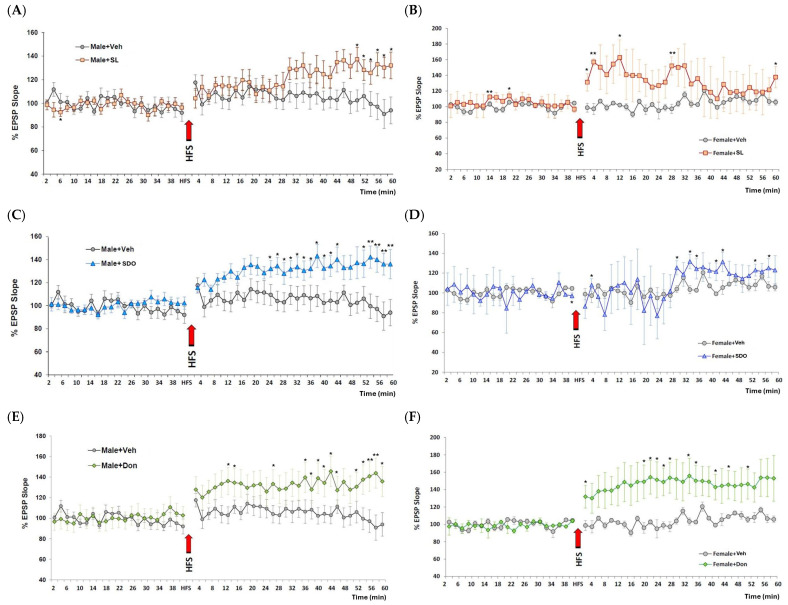
Analysis of hippocampal EPSP slopes and LTP induction in aged rats. The graphs illustrate the percentage of Excitatory Postsynaptic Potential (EPSP) slope before and after the induction of LTP via high-frequency stimulation (HFS, indicated by arrows) in: (**A**) Male + SL (n = 8, n = 7 in Male + Veh), (**B**) Female + SL (n = 3, n = 5 in Female + Veh), (**C**) Male + SDO (n = 8, n = 7 in Male + Veh), (**D**) Female + SDO (n = 4, n = 5 in Female + Veh), (**E**) Male + Don (n = 7), and (**F**) Female + Don (n = 3, n = 5 in Female + Veh). Data are presented as mean ± SEM. Statistical significance was determined by using independent-samples *t*-test. * *p* < 0.05, ** *p* < 0.01 compared to the respective Veh groups.

A profound therapeutic effect on synaptic plasticity was observed in the natural aging model. As expected, aged male control rats exhibited a significant impairment in LTP induction compared to what is typically observed in young animals. Remarkably, chronic 3-month treatment with both SL (10 mg/kg BW) and SDOs (100 mg/kg BW) robustly restored this age-related deficit. Following high-frequency stimulation (HFS), the magnitude of LTP in both SL- and SDO-treated aged male rats was significantly greater than that in aged Veh and comparable to the potentiation seen in the Don-treated positive control group (Figure 14A,C,E). This finding provides a strong mechanistic link between the observed cognitive enhancements and improved synaptic function in the aging male brain.

Consistent with the behavioral data on spatial memory, the LTP-enhancing effect of the silk-derived compounds was found to be sex-specific. Aged female control rats also showed impaired LTP induction; however, unlike in males, chronic treatment with either SL or SDOs did not consistently improve the magnitude of LTP in aged female rats (Figure 14B,D). Only the positive control, Don, was able to significantly enhance LTP in the aging female hippocampus. This result further supports the observation of sexual dimorphism in the therapeutic response to SL and SDOs, particularly concerning hippocampal-dependent functions.

Beyond direct antioxidant effects, the cognitive-enhancing efficacy of these bioactives is likely supported by their impact on systemic amnestic triggers. Our research team has previously shown that SDOs and SL mitigate major modifiable risk factors for dementia, including hypertension and hyperglycemia [22,24]. SDOs, in particular, enhance the eNOS/NO/sGC pathway, which is vital for maintaining cerebral blood flow and the health of the neurovascular unit [24]. Since cognitive function is highly dependent on consistent cerebral perfusion and glucose regulation, the dual-functional inhibition of ACE and DPP-IV by SDOs [25] may provide an indirect basis for the improved memory retention observed in our aging models. This suggests that SL and SDOs do not merely target a single pathogenic pathway but rather enhance synaptic resilience by addressing the multifactorial nature of brain aging.

The final sample size for the aged female groups, Female + SDO (n = 4), Female + Veh and Female + SL (n=3), reflects the inherent biological and technical challenges associated with longitudinal geriatric research. This study utilized 20-month-old Sprague-Dawley rats, a senescent age at which rats are highly prone to spontaneous pathologies and natural attrition [68]. Over the course of the 3-month chronic administration period, several animals were excluded due to the development of age-related tumors or health decline, exacerbated by the cumulative physiological stress of daily oral gavage [69]. Furthermore, the terminal in vivo LTP procedure requires prolonged, deep anesthesia, to which elderly rats exhibit significantly higher sensitivity and increased mortality rates compared to younger animals. To maintain the highest standards of data integrity, we applied strict inclusion criteria: only animals that maintained stable physiological parameters and a consistent electrophysiological baseline for the duration of the recording were included in the final analysis [70]. While the resulting sample size is small, the observed effect sizes provide a conservative but clear proof-of-concept regarding the synaptoprotective potential of the treatment in the aging female brain.

The strategic employment of two complementary rodent models, the $A\beta_{25-35}$-induced ICR mice and the naturally aged Sprague-Dawley rats, provides a basis for the distinct and overlapping efficacy highlighted in this study. While the ICR mice served as an efficient model for acute neurotoxicity, demonstrating that cognitive deficits can be triggered by specific toxic insults even without overt apoptosis, the aged rat model offered a more complex physiological landscape. This dual approach simulated both the acute and chronic multi-factorial decline of the human brain, enabling high-quality in vivo electrophysiological (LTP) and psychomotor assessments that would be technically limited in a mice-only study [71].

The efficacy of these bioactives encompasses two distinct pharmacological profiles. Their ability to prevent memory impairment in the A*β*-induced model demonstrates a robust neuroprotective effect against proteotoxic stress. In addition, their ability to significantly increase the magnitude of LTP and improve memory indices in aged rats, beyond the levels of age-matched controls, suggests a nootropic or cognitive-enhancing activity that directly modulates synaptic efficiency. Conversely, their distinct profiles are revealed through their specific physiological impacts and potencies: SL demonstrated high-potency antioxidant protection at a lower dose (10 mg/kg BW) due to its lipophilic nature, while SDOs (100 mg/kg BW) likely engaged broader systemic pathways to achieve comparable cognitive enhancement. This distinction was further underscored in the aging model, where the LTP-enhancing effects were notably sex-specific, showing robust restoration of synaptic plasticity in aged males but limited impact in aged females, even though both sexes exhibited improved recognition memory. Ultimately, the consistency of results across these dual models confirms that while SL and SDOs utilize different molecular entry points, they converge on a shared synaptoprotective mechanism, supporting their potential as multi-target candidates for mitigating both Aβ-induced and age-related cognitive decline.

This study allows for a unique comparative analysis of two distinct bioactives from a single source. The overlapping efficacy of both SL and SDOs in protecting against A*β*-induced deficits and improving cognitive function in aged animals points towards a shared, fundamental mechanism. This is strongly supported by recent biochemical characterizations demonstrating the potent and stable radical scavenging activities of both SDOs and SL (Table 1), with the latter showing superior antioxidant capacity to commercial free lutein [33]. This shared antioxidant prowess is likely the primary defense against the oxidative insult that underpins both A*β* toxicity and the aging process. Furthermore, the capacity of SDOs and SL to exert antihypertensive effects [21,23,35] suggests a potential overlapping benefit in promoting cerebrovascular health, a critical factor in maintaining cognitive function.

However, their distinct physicochemical natures, a lipophilic carotenoid (SL) versus hydrophilic peptides (SDOs), suggest complementary and potentially synergistic modes of action. As a lipophilic molecule, SL is ideally suited to integrate into neuronal membranes, where it can directly neutralize lipid peroxidation at the primary site of oxidative damage. This mechanism is critical for protecting against A*β*-induced toxicity, as demonstrated by the ability of lutein to mitigate oxidative stress and apoptosis in neuronal cell lines [30]. On the contrary, the hydrophilic SDOs are well-suited to operate in the aqueous cytosolic environment and the bloodstream. They can engage in broader cell signaling pathways and exert systemic benefits that indirectly support brain health. The known dual ACE and DPP-IV inhibitory activities of SDO [25] provide a powerful systemic mechanism. By improving blood pressure [21,24,35] and glycemic control [22], SDOs likely mitigate chronic cerebral hypoperfusion and systemic inflammation, two major contributors to age-related cognitive decline. The robust effect of both compounds on LTP in aged males, despite these different initial sites of action, further suggests a convergence on downstream pathways that regulate synaptic plasticity.

While the A*β* model is a widely accepted standard for screening agents against specific neurotoxic insults, the natural aging model offers distinct advantages in terms of translational relevance. It allows for the evaluation of bioactives within a complex physiological environment characterized by chronic oxidative stress, hormonal shifts, and systemic metabolic changes that more closely mirror human brain aging. Nevertheless, the landscape of neurodegeneration is vast. Future research could utilize transgenic AD models (e.g., 5xFAD) to assess the impact of silk bioactives on long-term plaque deposition [72], or tauopathy models to investigate their role in stabilizing the neuronal cytoskeleton [73]. Additionally, vascular dementia paradigms, such as chronic cerebral hypoperfusion [74], could further elucidate the vasorelaxant benefits of SDOs in maintaining cerebral blood flow. Utilizing these diverse models will be essential to fully map the multi-target potential of these *Bombyx mori* derivatives in promoting healthy brain aging.

The study provides the first comprehensive, comparative evidence that two distinct classes of bioactive compounds, SL and SDOs, derived from a single sustainable source, yellow silk cocoons, exert significant cognitive-enhancing effects and are associated with improved synaptic plasticity. Our findings demonstrate that chronic oral administration of both SL and SDOs effectively ameliorates deficits in recognition and spatial memory. Mechanistically, these behavioral improvements, particularly in the context of natural aging, are strongly associated with an enhancement of hippocampal synaptic plasticity. Collectively, these results highlight the potential of upcycling this agro-industrial byproduct into high-value nutraceuticals for promoting cognitive health.

Despite the significant findings, this study has certain limitations that warrant consideration. First, the sample size for the in vivo LTP recordings in aged female rats was relatively small due to natural attrition and age-related health complications. Second, while the behavioral and electrophysiological results strongly imply neuroprotective action, direct pharmacokinetic data, specifically regarding the blood–brain barrier permeability of the specific bioactives, was not established in this study. Further studies are needed to explore the long-term safety and efficacy of these compounds in human clinical trials to validate their potential as evidence-based nutraceuticals for age-related cognitive decline.

## 3. Materials and Methods

### 3.1. Evaluation of Radical Scavenging Potency, Reducing Power, and Metal-Chelating Capacity of SDOs and SL

#### 3.1.1. ABTS Radical Scavenging Activity Analysis

The antioxidant capacities of SDOs and SL were evaluated using a modified ABTS radical cation (${\mathrm{ABTS}}^{+}$) decolorization assay based on previously described methods of Laosam et al. (2024) [75] and Manupa et al. (2023) [33], respectively. The ${\mathrm{ABTS}}^{+}$ stock solution was prepared by reacting 7.0 mM ABTS (Sigma-Aldrich, Darmstadt, Germany) with potassium persulfate (2.45–2.60 mM) in the dark at room temperature for 12–16 h.

Due to the differing solubility profiles of the two bioactives, the working solutions were prepared separately: for SDO analysis, the stock was diluted with 50 mM phosphate buffer (pH 7.4) to reach an absorbance of 0.740±0.020 at 734 nm; for SL analysis, the stock was diluted with ethanol to achieve an absorbance of 0.700±0.020 at 734 nm [33]. For the SDO reaction, 20 µL of the sample was mixed with 1980 µL of the buffered working solution and incubated for 5 min in the dark. For the SL reaction, 10 µL of the extract was mixed with 1 mL of the ethanolic working solution. Absorbance was measured at 734 nm using a UV–Vis spectrophotometer (Amersham Biosciences, Ultrospec 6300 pro, Uppsala, Sweden). Trolox, BHA and tocopherol (Sigma-Aldrich, Saint Louis, MO, USA) were employed as standard antioxidants. The radical scavenging activity was calculated using the following equation:

(1) $$\mathrm{Scavenging}\;\mathrm{activity}\;(\%)=\left(\frac{A_{\mathrm{control}}-A_{\mathrm{sample}}}{A_{\mathrm{control}}}\right)\times 100$$

where $A_{\mathrm{control}}$ is the absorbance of the ${\mathrm{ABTS}}^{+}$ solution and $A_{\mathrm{sample}}$ is the absorbance of the test sample. The results are expressed as the half-maximal inhibitory concentration ($IC_{50}$), representing the concentration required to scavenge 50% of the ABTS radicals.

#### 3.1.2. Ferric Reducing Antioxidant Power Analysis

The ferric-reducing activities of SDOs and SL were determined using a modified FRAP assay based on previously established protocols [33,76,77]. The working FRAP reagent was prepared fresh by mixing 300 mM acetate buffer (pH 3.6), 10 mM 2,4,6-tripyridyl-s-triazine (TPTZ) in 40 mM HCl, and 20 mM ${\mathrm{FeCl}}_{3}\cdot 6H_{2}O$ in a 10:1:1 ratio (*v*/*v*).

For the SDO analysis, 100 µL of the sample was combined with 1 mL of the FRAP reagent and incubated at room temperature for 15 min. For the SL analysis, 10 µL of the extract was first diluted with 90 µL of distilled water before the addition of 900 µL of the FRAP reagent (pre-incubated at 37 °C for 5 min); the absorbance was then measured immediately and after a 4 min reaction period. In both cases, the absorbance was quantified at 593 nm using a UV–Vis spectrophotometer. For SDOs, Trolox (1 mg/mL) served as the reference standard. For SL, a standard curve was generated using ferrous sulfate (${\mathrm{FeSO}}_{4}$) in the range of 25–100 µM/mL, and results were compared against BHA and tocopherol. The reducing power was expressed as both the half-maximal inhibitory concentration ($IC_{50}$) and the FRAP value (µmol/L), calculated according to the following equation [36]:

(2) $$\mathrm{FRAP}\;\mathrm{value}=\left(\frac{A_{\mathrm{sample}}}{A_{\mathrm{control}}}\right)\times \mathrm{concentration}\;\mathrm{of}\;{\mathrm{Fe}}^{2+}\;\mathrm{standard}$$

where A represents the optical density (OD) at 593 nm.

#### 3.1.3. Metal Chelating Activity Analysis

The metal-chelating capacities of SDOs and SL were evaluated using a modified ferrozine-based assay according to previously established protocols [78]. This assay measures the ability of the bioactives to sequester ferrous ions ($Fe^{2+}$), thereby preventing the formation of the red-colored $Fe^{2+}$-ferrozine complex.

Due to their distinct chemical properties, the two bioactives were processed using specific solvent systems. For the SDO analysis, serial dilutions (1, 5, 10, 20, 30, and 40 mg/mL) were prepared in deionized water. A 50 µL aliquot of the sample was mixed with 1200 µL of deionized water and 25 µL of 2 mM $FeCl_{2}$ (Acros Organics, Bridgewater, NJ, USA), followed by a 3 min incubation in the dark. Subsequently, 50 µL of 5 mM ferrozine (Acros Organics, NJ, USA) was added, and the mixture was incubated for 20 min at room temperature in the absence of light. For the SL analysis, extracts were prepared in methanol at concentrations of 5, 10, and 15 µg/mL. A 0.5 mL aliquot of the sample was mixed with 100 µL of 0.6 mM $FeCl_{2}$ and 0.9 mL of methanol. After a 10 min incubation at room temperature, 0.1 mL of 5 mM ferrozine was added, followed by a further 5 min incubation. In both procedures, the absorbance was measured at 562 nm using a UV–Vis spectrophotometer. EDTA (0.31 mg/mL) (Sigma-Aldrich, Darmstadt, Germany) was employed as a positive control standard. The metal-chelating activity was calculated using the following equation:

(3) $$\mathrm{Inhibition}\;(\%)=\left(\frac{A_{\mathrm{control}}-A_{\mathrm{sample}}}{A_{\mathrm{control}}}\right)\times 100$$

where $A_{\mathrm{control}}$ represents the absorbance of the control (containing all reagents except the bioactive) and $A_{\mathrm{sample}}$ represents the absorbance of the test sample. Results are expressed as the half-maximal inhibitory concentration ($IC_{50}$).

### 3.2. Preparation and Characterization of Test Compounds

SDOs and SL were prepared from the yellow silk cocoons of *Bombyx mori* as detailed in Tocharus et al. (2024) [21] and Manupa et al. (2023) [33], respectively. The detailed pilot-scale production, peptide characterization, and stability analysis of the SDOs used in this study have been previously described [26]. Briefly, SDOs are a lyophilized powder of peptides with a molecular weight of <5 kDa. For oral administration, SDOs were freshly dissolved in distilled water each day. SL is previously characterized with details in previous studies of Manupa et al. (2023), Promphet et al. (2014), and Tocharus & Sutheerawattananonda (2026) [33,35,79]. For administration, SL was prepared as a suspension in its vehicle, 1% Tween 80 (Sigma-Aldrich, MO, USA) in 0.5% Dimethyl sulfoxide (DMSO) (Sigma-Aldrich, Darmstadt, Germany). The microbial safety of these extracts was confirmed during the characterization phase, ensuring they were free from pathogenic contamination including *Salmonella* spp. and *Escherichia coli* prior to animal administration [21,35]. Donepezil hydrochloride (Sigma-Aldrich, MO, USA) was used as a positive control in the aging study and was dissolved in distilled water. A*β* peptide fragment 25–35 (A*β*_25–35_) (Sigma-Aldrich, Darmstadt, Germany) was dissolved in sterile 0.9% saline. It was then aggregated by incubating at 37 °C for 96 h to form oligomeric species. This solution was stored at −20 °C until use, following a method adapted to induce neurotoxicity [44].

### 3.3. Animals

All experimental procedures were conducted in strict accordance with the Guide for the Care and Use of Laboratory Animals (8th ed., NIH, USA) and were approved by the Institutional Animal Care and Use Committee of Naresuan University (Protocol No. NU-AE550305). Forty-eight male ICR mice weighing 30–35 g at 4 weeks of age were obtained from the National Laboratory Animal Center, Mahidol University, Thailand. Fifty-six aged male and female Sprague-Dawley rats weighing 300–680 g at 20 months of age were also sourced for the natural aging study. Animals were housed in a temperature-controlled room (25 ± 2 °C) under a standard 12 h light/dark cycle with ad libitum access to standard rodent chow and water. All animals were acclimatized for at least one week before the start of experiments. Animals were randomly assigned to experimental groups using a weight-balancing allocation method following the ARRIVE guidelines [80]. Specifically, animals were individually identified by tail marking, and their initial body weights were recorded to ensure that the mean body weight was similar across all treatment groups at the start of the study. While experimenters were aware of treatment allocations during the study, all behavioral and electrophysiological data were analyzed using automated criteria and standardized software protocols to ensure reporting transparency and minimize experimental bias. To support scientific reproducibility, this research was conducted in accordance with the ARRIVE Essential 10 criteria. A completed Compliance Questionnaire is included as Supplementary File.

### 3.4. Paradigm 1: Evaluation of the Therapeutic Efficacy and Neuroprotective Mechanisms of SL and SDOs Against $A\beta_{25-35}$-Induced Cognitive Impairment

This paradigm was designed to evaluate the therapeutic efficacy of SL (10 mg/kg BW/day) and SDOs (100 mg/kg BW/day) administered chronically via oral gavage for 8 weeks against $A\beta_{25–35}$-induced cognitive impairment. Male ICR mice were divided into 4 groups (n = 12 per group): (1) Saline (NaCl 0.9%) + Veh (1% Tween 80 in 0.5% DMSO); (2) Aβ-induced (Aβ + Veh); (3) Aβ + SL 10 mg/kg BW; and (4) Aβ + SDO 100 mg/kg BW. The selection of dosages for SL (10 mg/kg BW) and SDOs (100 mg/kg BW) was based on preliminary dose–response screening studies conducted in our laboratory, which evaluated a range of concentrations (5–20 mg/kg BW for SL and 1–100 mg/kg BW for SDOs). From these initial assessments, SL at 10 mg/kg was identified as the lowest effective dose that consistently mitigated cognitive deficits in the A*β*-induced model. For SDOs, the 100 mg/kg BW dose demonstrated the highest therapeutic efficacy, providing robust and comprehensive protection across multiple memory domains, including both object recognition and spatial navigation. Thus, these specific concentrations were utilized in the current chronic administration paradigm to further investigate their long-term neuroprotective potential.

ICR mice were selected as a standard for pharmacological screening due to their predictable sensitivity to $A\beta_{25-35}$ [81,82], which induces reproducible cognitive deficits in NOR and MWM assays. This approach allowed for efficient validation of neuroprotective efficacy before proceeding to the more complex physiological assessments in the rat aging model.

The study commenced with daily oral gavage of vehicle, SL, or SDOs for 8 weeks. Baseline cognitive functions were assessed over a 2-week period using the MWM, including probe trials, and the NOR test. Subsequently, memory impairment was induced via i.c.v. injection of $A\beta_{25–35}$ peptides in the three experimental groups, while the control group received i.c.v. saline.

#### 3.4.1. Intracerebroventricular Surgery

**Figure 15:**
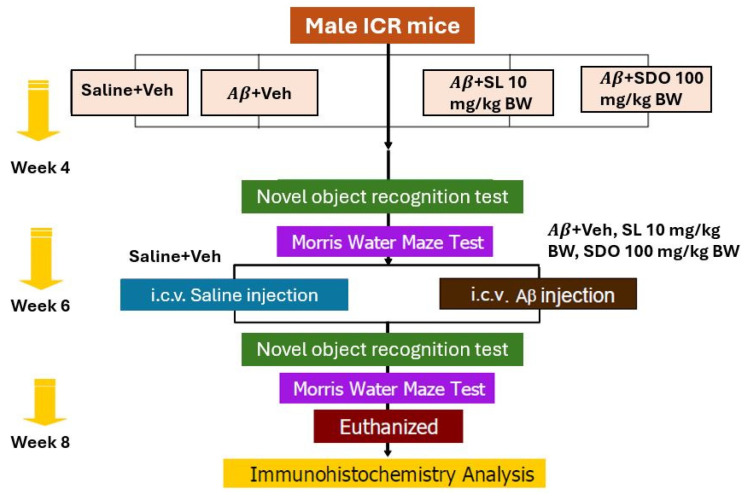
Experimental grouping and schematic protocol for the Aβ-induced amnesia model in male ICR mice.

For the A*β*-induced amnesia models, mice were injected with pentobarbital sodium (Nembutal™; CEVA Animal Health Ltd., Ontario, CA, Canada) at a dose of 40 mg/kg BW via intraperitoneal injection (i.p.). Its head was cleaned with 70% alcohol after complete anesthesia. An i.c.v. administration of A*β*_25–35_ peptides was performed using a free-hand method as previously described by Nakdook et al. (2010) [83]. A 10 µL Hamilton syringe with 26-gauge stainless-steel needle 2.4 mm long. The needle was inserted unilaterally 1 mm to the right of the midline point equidistant from each eye, at an equal distance between the eyes and the ears, and perpendicular to the plane of the skull (−0.5 mm anterior/posterior, +1.0 mm medial/lateral, and −2.4 mm dorsal/ventral) [84,85]. The depth was strictly controlled by the 2.4 mm needle length, ensuring the tip reached the lateral ventricle upon full insertion to the syringe hub. Aggregated peptides (10 μg in 10 μL saline) or sterile saline were delivered gradually within 30 s into the right lateral ventricle, and the needle was left in place for 1 min to ensure optimal diffusion and prevent backflow before being slowly withdrawn. The accuracy of the injection placement was verified post-mortem by visual inspection of the needle track during brain dissection [86]. All mice were allowed a recovery period of at least 4 days before subsequent behavioral testing. Figure 15 represents experimental grouping and schematic protocol for the Aβ-induced amnesia model in male ICR mice.

#### 3.4.2. Histology and Immunohistochemistry for Apoptosis Markers in ICR Mice

The study aimed to elucidate the underlying neuroprotective mechanisms by investigating whether the amelioration of memory deficits is associated with the prevention of hippocampal neuronal death and the modulation of apoptosis-related protein expression. Upon completion of the behavioral assessments, all mice were euthanized via an anesthetic overdose, and the brain tissues were rapidly harvested. The brains were fixed in 4% paraformaldehyde (PFA), followed by standard histological processing and paraffin embedding. Coronal sections of the hippocampus were prepared at a thickness of 10 μm.

To evaluate apoptotic cell death, DNA fragmentation was detected using a terminal deoxynucleotidyl transferase dUTP nick end labeling (TUNEL) assay (FragEL™ DNA Fragmentation Detection Kit; Sigma-Aldrich, Darmstadt, Germany). Briefly, the sections were incubated in the TUNEL reaction mixture at 37 °C for 1 h and subsequently rinsed in phosphate-buffered saline (PBS). The reaction was visualized using 3,3′-diaminobenzidine (DAB) as the chromogen, followed by counterstaining with methyl green. The presence of TUNEL-positive cells and general neuronal morphology were qualitatively assessed by a pathologist under a light microscope. Hippocampal subregions, CA1, were evaluated for signs of neuronal damage, such as pyknotic nuclei or overt neurodegeneration, following standard histological guidelines [87]. While the pathologist was aware of treatment allocations, the assessment focused on identifying consistent pathological features across multiple sections per animal.

To assess apoptosis, sections were stained for key apoptotic and anti-apoptotic proteins, including Bax and Bcl-2, using standard immunohistochemical protocols. Briefly, sections were incubated with primary antibodies (anti-Bax, anti-Bcl-2; Abcam, Cambridge, UK) [88], followed by a biotinylated secondary antibody and visualized using DAB substrate kit.

### 3.5. Paradigm 2: Natural Aging Model in Rats

This paradigm evaluated the therapeutic effects of SL and SDOs on age-related cognitive decline. Aged male and female Sprague-Dawley rats were divided into four groups each (n = 6–8): (1) Aged + Veh, (2) Aged + SL (SL 10 mg/kg BW/day orally), (3) Aged + SDO (SDO 100 mg/kg BW/day orally), (4) Aged + Don (Donepezil 1 mg/kg BW/day orally). Treatments were administered daily for 3 months, during which behavioral, psychomotor, and electrophysiological tests were conducted.

**Figure 16:**
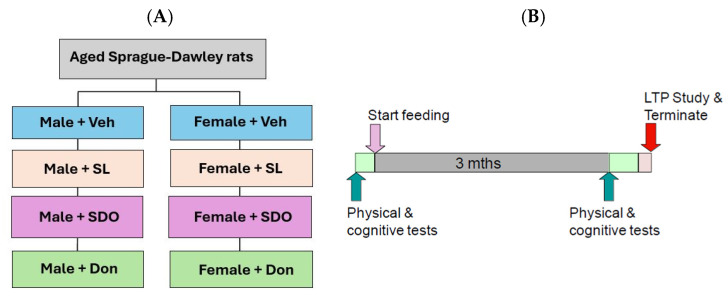
Experimental design and assessment protocols: (**A**) Classification of aged rats into male and female, (**B**) sequential steps for evaluating the effects of lutein extract and oligopeptides on physical performance and cognitive functions (learning and memory).

Sprague-Dawley rats were selected for the natural aging model due to their physiological and behavioral complexity, which more closely mirrors the multi-factorial nature of human aging [89,90]. Technically, the larger rat brain was essential for the precision required in in vivo hippocampal LTP recordings [71], allowing for stable electrode placement in the CA1 region to capture high-quality synaptic plasticity data. Figure 16 represents experimental design and assessment protocols of the natural aging model in rats.

#### 3.5.1. Novel Object Recognition Test

The NOR test was used to assess recognition memory, a form of non-spatial memory. The procedure was adapted from Myhrer (1988) [91]. The apparatus consisted of an open-field box (120 × 120 cm). The test involved three phases: habituation, training (familiarization), and testing. During the training phase, two identical objects (A and B) were placed in the box, and a mouse was allowed to explore for 5–10 min. After a retention interval, the animal was returned to the box during the testing phase, where one of the familiar objects was replaced with a novel object (C). The time spent exploring each object was recorded. A Recognition Index (RI) was calculated as follows [92]:

RI = [Time (Novel)/(Time (Novel) + Time (Familiar))] × 100(4)

#### 3.5.2. Morris Water Maze Test

Spatial learning and memory were assessed using the MWM test, following the protocol described by Morris (1984) [93]. The maze consisted of a circular pool (100 cm diameter) filled with opaque water. During the acquisition phase (learning trials), mice were trained for 7–10 consecutive days to find a hidden platform submerged beneath the water surface. The time taken to locate the platform (escape latency) was recorded. For the probe trial, conducted 24 h after the last training session, the platform was removed, and the animals were allowed to swim for 60 s. The time spent in the target quadrant (where the platform was previously located) was measured as an index of spatial memory retention [94].

#### 3.5.3. Open-Field Test

General locomotor activity and exploratory behavior were assessed before treatment (Pre-Rx) and after 3 months of treatment (Post-Rx). To ensure the animals were in their active state, testing was conducted with standard room lighting turned off; red light was utilized to provide visibility for the experimenter. Prior to the start of testing, rats were allowed to acclimate to the procedure room for a minimum of 30 min. Each subject was placed in the center of a square open-field maze measuring 50 cm (length) × 50 cm (width) × 38 cm (height), constructed from white high-density, non-porous plastic [95]. To remove scent clues left by previous subjects, the chamber was wiped with 95% ethanol and allowed to evaporate between each 5 min testing session. The total distance traveled was recorded and analyzed using Anymaze video tracking software (version 4.7, Stoelting Co., Wood Dale, IL, USA).

#### 3.5.4. Beam Walking Test

To assess age-related decline in motor coordination and balance, beam walking tests were performed by training rats to traverse a narrow wooden beam (1.5 cm wide, 200 cm long). The time taken to cross the beam (walking time) and any instances of falling were recorded [96].

#### 3.5.5. Grip Strength Test

To assess forelimb grip strength, rats were allowed to grasp a metal wire bar with both forepaws. The researcher then gently pulled the rat backward by its body until it released its grip. This procedure was performed in three consecutive trials. The grip force was converted into an electrical signal using an isometric force transducer (Grass Technologies, model FT03, West Warwick, RI, USA) [97] connected to a Bridge-Amp (model ML101, ADInstruments, Sydney, Australia) and a PowerLab 4/25T data acquisition system (ADInstruments, Sydney, Australia). The force was displayed as tension (measured in grams) and recorded as a line graph using Chart software version 5.4 (ADInstruments, Sydney, Australia).

#### 3.5.6. In Vivo Electrophysiology: Long-Term Potentiation

To assess synaptic plasticity, in vivo field potential recordings were performed in the CA1 region of the hippocampus in anesthetized rats (urethane, 1.5 g/kg BW, i.p.). A tungsten recording microelectrode was stereotaxically positioned into the CA1 pyramidal cell layer of the hippocampus (approximately 2.0 mm ventral to the brain surface). For stimulation, a steraide, concentric bipolar electrode (model NE-100, Rhodes Medical Instruments, Inc. Carpinteria, CA, USA) was implanted into the Schaffer collateral (Sch) pathway.

The recorded neuronal signals were amplified using a microelectrode amplifier (Model 1800, A-M Systems, Sequim, WA, USA) and digitized via a PowerLab 4SP data acquisition system (ADInstruments, Sydney, Australia). Data were visualized and processed using Chart Pro software (v5.5, ADInstruments, Sydney, Australia). The evoked field potentials, specifically population excitatory postsynaptic potentials (pEPSPs), were elicited by low-frequency stimulation of the CA3 Schaffer collateral projections to CA1 pyramidal cells at a rate of one pulse every 2 min. Electrophysiological responses were quantified by measuring the pEPSP amplitude and the initial slope of the potential, with data expressed as a percentage of the baseline control period [65,98]. Following the baseline recording, high-frequency stimulation (HFS) was applied to induce synaptic plasticity using the following parameters: 10 trains of pulses delivered at 2 s inter-train intervals, with each train consisting of 20 pulses at 200 Hz.

### 3.6. Statistical Analysis

Statistical analyses were performed using SigmaStat (version 3.0, SPSS Inc., Armonk, NY, USA). For comparisons between two experimental groups, an independent-sample t-test was employed. As for the comparisons of the same group of pre- and post-treatment, a paired *t*-test was used. For multiple group comparisons, one-way analysis of variance (ANOVA) was performed, followed by Dunnett’s multiple comparison tests. A comprehensive statistical report can be found in Supplementary File.

## 4. Conclusions

This study provides the first direct comparative evidence that SL and SDOs, upcycled from underutilized yellow silk cocoons of *Bombyx mori*, are potent agents that exhibit both neuroprotective properties by mitigating acute neurotoxicity and nootropic activity and by restoring and enhancing hippocampal synaptic plasticity in the aging model. Our investigation across acute neurotoxicity and chronic aging models demonstrates that these compounds protect against A*β*-induced synaptic failure and improve age-related cognitive decline, primarily by enhancing hippocampal synaptic plasticity. This study positions SL and SDOs as ideal candidates for early interventions to bolster synaptic resilience before widespread neuronal death occurs by focusing on synaptoprotective strategy. Their distinct chemical natures, a lipid-soluble carotenoid and water-soluble peptides, suggest a multi-pronged therapeutic approach targeting both membrane-level oxidative stress and cellular signaling pathways. Beyond the neuroscientific implications, the findings provide a powerful proof-of-concept for the valorization of agro-industrial byproducts, transforming sericulture waste into high-value, evidence-based nutraceuticals. In addition, this waste-to-wellness paradigm offers a sustainable, cost-effective avenue for developing novel health products while creating new economic opportunities. Ultimately, our study lays a critical scientific foundation, paving the way for the future clinical development of silk-derived bioactives as safe and effective agents to promote healthy brain aging and address the global challenge of cognitive decline.

## Figures and Tables

**Table 1:** Comparative antioxidant activities ($IC_{50}$) of SDOs and SL.

Antioxidant Assay	SDOs (μg/mL)	SL (μg/mL)
ABTS	2100.00±0.07	324.15±10.8 ^1^
FRAP	9070.00±0.07	30.33±0.06 ^1^
Metal chelating	22,850.00±0.07	Not detected

Data are expressed as mean ± SD. ^1^ Manupa et al. (2023) [33].

**Table 2:** Body weights of aged male and female rats before and after 3 months of daily oral administration of SL, SDOs, or Don.

Treatment	Sex	n	Pre-Treatment BW (g)	Post-Treatment BW (g)	Change (%)
**Veh**	Male	7	626.8 ± 15.6	614.3 ± 15.3	−2.0
	Female	6	356.4 ± 26.1	363.6 ± 27.2	+2.0
**SL**	Male	8	655.0 ± 27.9	624.1 ± 31.1 **	−4.7
	Female	7	332.0 ± 17.0	347.0 ± 23.4 **	+4.5
**SDO**	Male	8	623.2 ± 14.3	617.8 ± 23.1	−0.9
	Female	7	351.0 ± 19.8	367.1 ± 4.6 **	+4.6
**Don**	Male	7	610.5 ± 13.0	622.2 ± 30.5	+1.9
	Female	6	362.8 ± 30.1	385.8 ± 42.7 *	+6.3

Data are presented as mean ± SEM. Statistical significance was determined using a paired sample *t*-test to compare pre-treatment and post-treatment values within each group. * *p* < 0.05, ** *p* < 0.01 compared to pre-treatment.

## Data Availability

The original contributions presented in this study are included in the article/Supplementary Material. Further inquiries can be directed to the corresponding author.

## Supplementary Materials

The following supporting information can be downloaded at: https://www.mdpi.com/article/10.3390/ijms27072986/s1.
