# Supplementary material for: Distinct and Overlapping Neuroprotective Efficacy of Silk Lutein and Sericin-Derived Oligopeptides from Yellow Silk Cocoons in Rodent Models of Aβ-Induced and Age-Related Cognitive Decline
Source: Int J Mol Sci. 2026 Mar 25;27(7):2986. doi: 10.3390/ijms27072986 (PMC13072934; doi:10.3390/ijms27072986)
Supplement: Supplementary file 1 [file ijms-27-02986-s001.zip › ijms-4155740-supplementary file.pdf]

Statistical reports

Figure S1. Effect of chronic administration of SL and SDO on body weight in the Aβ_{25-35}-induced amnesia model.

ANOVA	Dgree of freedom (DF)	F-value	p-value	Significance
Week 1	(3, 36)	6.963	0.001	Significant
Week 2	(3, 36)	5.120	0.005	Significant
Week 3	(3, 44)	1.324	0.279	Not significant (NS)
Week 4	(3, 44)	1.287	0.291	NS
Week 5	(3, 44)	0.465	0.708	NS
Week 6	(3, 44)	0.187	0.905	NS
Week 7	(3, 44)	0.607	0.614	NS
Week 8	(3, 44)	0.431	0.732	NS
Week 9	(3, 44)	1.141	0.343	NS
Week 10	(3, 44)	0.515	0.674	NS

Dunnett multiple comparison	Aβ+SL vs Aβ+Veh	Aβ+SDO vs Aβ+Veh	Saline+Veh vs Aβ+Veh
Week 1	0.072	0.874	0.001##
Week 2	0.842	0.022#	0.961
Week 3	0.969	0.327	1.000
Week 4	0.455	0.883	0.993
Week 5	0.811	0.979	0.985
Week 6	0.935	0.998	0.994
Week 7	0.612	0.994	0.990
Week 8	0.807	1.000	0.965
Week 9	0.276	0.345	0.324
Week 10	0.702	0.999	0.655

#$p < 0.05$, ##$p < 0.01$ compared Aβ+Veh group.

Dunnett multiple comparison	Aβ+Veh vs. Saline+Veh	Aβ+SL vs. Saline+Veh	Aβ+SDO vs. Saline+Veh
Week 1	0.001**	0.168	0.003**
Week 2	0.961	0.985	0.008**
Week 3	1.000	0.953	0.357
Week 4	0.993	0.333	0.964
Week 5	0.985	0.626	1.000
Week 6	0.994	0.838	1.000
Week 7	0.990	0.448	0.943
Week 8	0.965	0.554	0.966
Week 9	0.324	0.999	1.000
Week 10	0.655	1.000	0.726

** $p < 0.01$ compared Saline+Veh group.

Table S2. Body weights of aged male and female rats before and after 3 months of daily oral administration of SL, SDO, or Don.

Paired-samples t-test	DF	t-value	p-value	Significance
Male+Veh Before vs After	5	1.324	0.122	NS
Female+Veh Before vs After	4	-1.037	0.179	NS
Male+SL Before vs After	6	4.594	<0.01	**
Female+SL Before vs After	6	-4.910	<0.01	**
Male+SDO Before vs After	5	0.764	0.2395	NS
Female+SDO Before vs After	5	-4.223	<0.01	**
Male+Don Before vs After	4	-1.621	0.090	NS
Female+Don Before vs After	5	-2.389	0.031	*

*$p < 0.05$, **$p < 0.01$ compared post-treatment to pre-treatment values within each group.

Figure S2. Protective effects of SL and SDO on recognition memory in the $A\beta_{25-35}$-induced amnesia model.

Paired-samples t-test	DF	t-value	p-value	Significance

Saline+Veh object A vs object B	13	-1.537	0.074	NS
Saline+Veh object A vs object C	13	-9.363	<0.01	**
Aβ+Veh object A vs object B	10	0.524	0.306	NS
Aβ+Veh object A vs object C	10	-0.303	0.384	NS
Aβ+SL object A vs object B	6	0.037	0.486	NS
Aβ+SL object A vs object C	6	-7.049	<0.01	**
Aβ+SDO object A vs object B	10	-1.200	0.129	NS
Aβ+SDO object A vs object C	10	-6.294	<0.01	**

**$p < 0.01$ compared to object A within each group.

Figure S3. Comparison of mean escape latencies across experimental groups during the training phase in $A\beta_{25-35}$-induced amnesia model.

ANOVA	DF	F-value	p-value	Significance
Day 1	(3, 125)	2.724	0.047	Significant
Day 2	(3, 125)	4.009	0.009	Significant
Day 3	(3, 125)	0.370	0.775	NS
Day 4	(3, 125)	2.914	0.037	Significant
Day 5	(3, 125)	2.260	0.085	NS
Day 6	(3, 125)	0.781	0.507	NS
Day 7	(3, 125)	0.854	0.467	NS
Day 8	(3, 125)	0.089	0.966	NS
Day 9	(3, 122)	0.497	0.685	NS
Day 10	(3, 122)	0.253	0.859	NS
Day 11	(3, 122)	2.787	0.044	Significant
Day 12	(3, 122)	1.349	0.262	NS
Day 13	(3, 122)	1.557	0.203	NS
Day 14	(3, 119)	2.002	0.117	NS
Day 15	(3, 122)	1.255	0.293	NS
Day 16	(3, 122)	0.631	0.596	NS
Day 17	(3, 122)	0.967	0.411	NS

Dunnett multiple comparison	Aβ+SL vs Aβ+Veh	Aβ+SDO vs Aβ+Veh	Saline+Veh vs Aβ+Veh
Day 1	1.000	0.051	1.000
Day 2	0.978	0.350	0.025#

Day 3	0.835	0.609	0.918
Day 4	0.135	0.873	0.326
Day 5	0.078	1.000	0.630
Day 6	0.926	0.637	1.000
Day 7	0.762	0.332	0.482
Day 8	0.998	0.966	0.947
Day 9	0.950	0.670	0.584
Day 10	0.958	0.966	0.723
Day 11	0.043#	0.144	0.995
Day 12	0.635	0.830	0.624
Day 13	0.155	0.171	0.617
Day 14	0.085	0.620	1.000
Day 15	0.206	0.999	0.919
Day 16	0.638	0.803	0.990
Day 17	0.433	0.517	1.000

#$p < 0.05$ compared Aβ+Veh group.

**Dunnett multiple comparison**	**Aβ+Veh vs Saline+Veh**	**Aβ+SL vs Saline+Veh**	**Aβ+SDO vs Saline+Veh**
Day 1	1.000	1.000	0.090
Day 2	0.024*	0.480	0.967
Day 3	0.904	0.999	0.948
Day 4	0.303	0.985	0.106
Day 5	0.597	0.619	0.568
Day 6	1.000	0.922	0.692
Day 7	0.451	0.916	0.999
Day 8	0.937	0.972	0.999
Day 9	0.552	0.829	0.993
Day 10	0.693	0.920	0.906
Day 11	0.994	0.108	0.272
Day 12	0.591	0.999	0.229
Day 13	0.584	0.812	0.844
Day 14	1.000	0.117	0.640
Day 15	0.906	0.561	0.946

Day 16	0.988	0.501	0.656
Day 17	1.000	0.454	0.532

*$p < 0.05$ compared Saline+Veh group.

Figure S4. Spatial memory performance during the probe trial test, represented by the percentage of time spent in each zone for $A\beta_{25-35}$-induced amnesia model.

Paired-samples t-test	DF	t-value	p-value	Significance
Saline+Veh Zone 1 vs Zone 4 (Pre-injection)	11	-4.888	<0.01	**
Saline+Veh Zone 2 vs Zone 4 (Pre-injection)	11	-3.074	<0.01	**
Saline+Veh Zone 3 vs Zone 4 (Pre-injection)	11	-3.272	<0.01	**
Saline+Veh Zone 1 vs Zone 4 (Post-injection)	8	-12.267	<0.01	**
Saline+Veh Zone 2 vs Zone 4 (Post-injection)	8	-14.474	<0.01	**
Saline+Veh Zone 3 vs Zone 4 (Post-injection)	8	-3.765	<0.01	**
Aβ +Veh Zone 1 vs Zone 4 (Pre-injection)	11	-5.256	<0.01	**
Aβ +Veh Zone 2 vs Zone 4 (Pre-injection)	11	-3.765	<0.01	**
Aβ +Veh Zone 3 vs Zone 4 (Pre-injection)	11	-3.332	<0.01	**
Aβ +Veh Zone 1 vs Zone 4 (Post-injection)	8	-3.980	<0.01	**
Aβ +Veh Zone 2 vs Zone 4 (Post-injection)	8	-1.164	0.139	NS
Aβ +Veh Zone 3 vs Zone 4 (Post-injection)	8	-1.045	0.164	NS
Aβ +SL Zone 1 vs Zone 4 (Pre-injection)	11	-5.099	<0.01	**
Aβ +SL Zone 2 vs Zone 4 (Pre-injection)	11	-2.571	<0.01	**
Aβ +SL Zone 3 vs Zone 4 (Pre-injection)	11	-3.535	<0.01	**
Aβ +SL Zone 1 vs Zone 4 (Post-injection)	11	-3.666	<0.01	**
Aβ +SL Zone 2 vs Zone 4 (Post-injection)	11	-0.635	0.269	NS
Aβ +SL Zone 3 vs Zone 4 (Post-injection)	11	-3.789	<0.01	**
Aβ +SDO Zone 1 vs Zone 4 (Pre-injection)	11	-3.286	<0.01	**
Aβ +SDO Zone 2 vs Zone 4 (Pre-injection)	11	-2.540	<0.01	**
Aβ +SDO Zone 3 vs Zone 4 (Pre-injection)	11	-3.301	<0.01	**
Aβ +SDO Zone 1 vs Zone 4 (Post-injection)	11	-6.233	<0.01	**
Aβ +SDO Zone 2 vs Zone 4 (Post-injection)	11	-1.441	0.089	NS
Aβ +SDO Zone 3 vs Zone 4 (Post-injection)	11	-5.301	<0.01	**

**$p < 0.01$ compared to Zone 4 within each group and session.

Figure S8. Therapeutic effects of SL, SDO, and Don on the escape latency of male and female aged rats during a 1-week MWM learning trial.

Independent samples t-test	DF	t-value	p-value	Significance

Male+SL vs Male+Veh Day 1	8.881	-0.036	0.486	NS
Male+SL vs Male+Veh Day 2	8.583	1.159	0.139	NS
Male+SL vs Male+Veh Day 3	7.800	-1.405	0.099	NS
Male+SL vs Male+Veh Day 4	9.931	-0.451	0.331	NS
Male+SL vs Male+Veh Day 5	11.883	-1.590	0.069	NS
Male+SL vs Male+Veh Day 6	11.644	-0.949	0.181	NS
Male+SL vs Male+Veh Day 7	7.855	0.122	0.453	NS

**Independent samples t-test**	**DF**	**t-value**	**p-value**	**Significance**
Male+SDO vs Male+Veh Day 1	6.779	0.158	0.440	NS
Male+SDO vs Male+Veh Day 2	6.573	0.794	0.228	NS
Male+SDO vs Male+Veh Day 3	4.032	-0.983	0.191	NS
Male+SDO vs Male+Veh Day 4	5.332	-0.671	0.265	NS
Male+SDO vs Male+Veh Day 5	3.138	-1.641	0.098	NS
Male+SDO vs Male+Veh Day 6	5.326	-0.202	0.424	NS
Male+SDO vs Male+Veh Day 7	6.182	-1.294	0.121	NS

**Independent samples t-test**	**DF**	**t-value**	**p-value**	**Significance**
Male+Don vs Male+Veh Day 1	6.137	-1.793	0.061	NS
Male+Don vs Male+Veh Day 2	7.990	1.342	0.108	NS
Male+Don vs Male+Veh Day 3	3.226	-1.884	0.075	NS
Male+Don vs Male+Veh Day 4	7.684	0.731	0.244	NS
Male+Don vs Male+Veh Day 5	5.858	-0.587	0.290	NS
Male+Don vs Male+Veh Day 6	6.108	-1.062	0.164	NS
Male+Don vs Male+Veh Day 7	6.386	-0.438	0.338	NS

*p <0.05 compared to Male + Veh group.

**Independent samples t-test**	**DF**	**t-value**	**p-value**	**Significance**
Female+SL vs Female+Veh Day 1	8.982	1.464	0.089	NS
Female+SL vs Female+Veh Day 2	4.916	1.941	0.056	NS

Female+SL vs Female+Veh Day 3	7.638	0.337	0.373	NS
Female+SL vs Female+Veh Day 4	9.515	-0.536	0.302	NS
Female+SL vs Female+Veh Day 5	5.123	1.959	0.053	NS
Female+SL vs Female+Veh Day 6	8.348	0.645	0.268	NS
Female+SL vs Female+Veh Day 7	9.999	2.009	0.036	*

Independent samples t-test	DF	t-value	p-value	Significance
Female+SDO vs Female+Veh Day 1	7.602	0.006	0.498	NS
Female+SDO vs Female+Veh Day 2	8.430	-0.262	0.400	NS
Female+SDO vs Female+Veh Day 3	9.482	-0.028	0.489	NS
Female+SDO vs Female+Veh Day 4	9.582	1.302	0.112	NS
Female+SDO vs Female+Veh Day 5	9.233	1.366	0.102	NS
Female+SDO vs Female+Veh Day 6	5.890	1.760	0.065	NS
Female+SDO vs Female+Veh Day 7	6.736	-0.131	0.450	NS

Independent samples t-test	DF	t-value	p-value	Significance
Female+Don vs Female+Veh Day 1	8.351	0.424	0.341	NS
Female+Don vs Female+Veh Day 2	8.279	-0.088	0.466	NS
Female+Don vs Female+Veh Day 3	8.964	-0.140	0.446	NS
Female+Don vs Female+Veh Day 4	7.741	-1.087	0.155	NS
Female+Don vs Female+Veh Day 5	4.764	2.686	0.023	*
Female+Don vs Female+Veh Day 6	6.997	1.699	0.067	NS
Female+Don vs Female+Veh Day 7	8.985	2.637	0.014	*

*p <0.05 compared to Female + Veh group.

Figure S9. Comparison of spatial memory indices in aged male and female rats during the MWM probe trial.

Independent samples t-test	DF	t-value	p-value	Significance
Male+SL vs Male+Veh	9.355	-1.935	0.042	*
Male+SDO vs Male+Veh	4.760	-2.239	0.039	*
Male+Don vs Male+Veh	4.021	-2.305	0.041	*

*p <0.05 compared to Male + Veh group.

Independent samples t-test	DF	t-value	p-value	Significance
Female+SL vs Female+Veh	9.852	-0.626	0.273	NS
Female+SDO vs Female+Veh	7.027	-0.385	0.356	NS
Female+Don vs Male+Veh	8.615	-1.917	0.045	*

*p <0.05 compared to Female + Veh group.

Figure S10. Comparison of object recognition indices in aged male and female rats during Pre-Rx and Post-Rx phases.

Paired-samples t-test	DF	t-value	p-value	Significance
Male+Veh Pre-Rx vs Post-Rx	7	-0.881	0.204	NS
Male+SL Pre-Rx vs Post-Rx	6	-2.213	0.035	*
Male+SDO Pre-Rx vs Post-Rx	2	-1.033	0.205	NS
Male+Don Pre-Rx vs Post-Rx	3	0.651	0.281	NS

Paired-samples t-test	DF	t-value	p-value	Significance
Female+Veh Pre-Rx vs Post-Rx	7	-0.919	0.195	NS
Female+SL Pre-Rx vs Post-Rx	6	-2.208	0.035	*
Female+SDO Pre-Rx vs Post-Rx	6	-2.290	0.031	*
Female+Don Pre-Rx vs Post-Rx	5	-1.221	0.138	NS

*p <0.05 compared to Pre-Rx value within the same group.

Figure S11. Exploratory behavior and physical performance of aged male and female rats following administration of SL and SDO extracts from yellow silk cocoons, or Donepezil, assessed by the Open-field test.

Paired-samples t-test	DF	t-value	p-value	Significance

Male+Veh Pre-Rx vs Post-Rx	6	1.456	0.098	NS
Male+SL Pre-Rx vs Post-Rx	7	1.828	0.055	NS
Male+SDO Pre-Rx vs Post-Rx	3	0.296	0.393	NS
Male+Don Pre-Rx vs Post-Rx	3	1.966	0.072	NS

**Paired-samples t-test**	**DF**	**t-value**	**p-value**	**Significance**
Female+Veh Pre-Rx vs Post-Rx	4	3.009	0.020	*
Female+SL Pre-Rx vs Post-Rx	6	1.601	0.080	NS
Female+SDO Pre-Rx vs Post-Rx	6	1.302	0.121	NS
Female+Don Pre-Rx vs Post-Rx	5	-2.507	0.027	*

*p <0.05 compared to Pre-Rx value within the same group.

Figure S12. Motor coordination and physical performance of aged male and female rats assessed by the narrow beam walking test. Data compares the effects of Veh, SL, SDO, and Don at baseline (Pre-Rx) and following 3 months of treatment (Post-Rx).

**Paired-samples t-test**	**DF**	**t-value**	**p-value**	**Significance**
Male+Veh Pre-Rx vs Post-Rx	5	2.037	0.049	*
Male+SL Pre-Rx vs Post-Rx	7	2.487	0.021	*
Male+SDO Pre-Rx vs Post-Rx	3	6.880	0.003	**
Male+Don Pre-Rx vs Post-Rx	3	2.889	0.032	*

**Paired-samples t-test**	**DF**	**t-value**	**p-value**	**Significance**

Female+Veh Pre-Rx vs Post-Rx	4	3.288	0.015	*
Female+SL Pre-Rx vs Post-Rx	6	4.021	0.004	**
Female+SDO Pre-Rx vs Post-Rx	6	2.983	0.013	*
Female+Don Pre-Rx vs Post-Rx	5	3.746	0.007	**

*p <0.05, **p <0.01 compared to Pre-Rx value within the same group.

Figure S13. Forelimb grip strength of aged male and female rats before (Pre-Rx) and after 3 months (Post-Rx) of treatment with vehicle, SL, SDO, or Don.

Paired-samples t-test	DF	t-value	p-value	Significance
Male+Veh Pre-Rx vs Post-Rx	6	-0.189	0.428	NS
Male+SL Pre-Rx vs Post-Rx	7	-0.058	0.478	NS
Male+SDO Pre-Rx vs Post-Rx	4	-0.995	0.188	NS
Male+Don Pre-Rx vs Post-Rx	4	-0.077	0.472	NS

Paired-samples t-test	DF	t-value	p-value	Significance
Female+Veh Pre-Rx vs Post-Rx	4	2.424	0.036	*
Female+SL Pre-Rx vs Post-Rx	6	0.251	0.405	NS
Female+SDO Pre-Rx vs Post-Rx	6	0.477	0.325	NS
Female+Don Pre-Rx vs Post-Rx	5	-6.403	<0.01	**

*p <0.05, ** p <0.01 compared to Pre-Rx value within the same group.

Figure S14. Analysis of hippocampal EPSP slopes and LTP induction in aged male and female rats.

Independent samples t-test	DF	t-value	p-value	Significance
**Female+SL vs Female +Veh (Pre-HFS)**				
2 mins	2.027	0.118	0.4585	NS
4 mins	2.020	-0.520	0.3270	NS
6 mins	2.827	-0.614	0.2925	NS
8 mins	2.353	-0.674	0.2805	NS
10 mins	2.234	-0.012	0.4960	NS
12 mins	2.497	-1.133	0.1770	NS
14 mins	4.583	-4.603	<0.01	**
16 mins	2.795	-2.369	0.0525	NS
18 mins	2.626	-1.192	0.1650	NS
20 mins	3.137	-2.399	**0.0460**	*
22 mins	3.271	-0.598	0.2945	NS
24 mins	2.378	-1.040	0.1960	NS
26 mins	2.963	-1.373	0.1320	NS
28 mins	4.766	-0.380	0.3600	NS
30 mins	2.165	-0.677	0.2815	NS
32 mins	2.464	-0.711	0.2690	NS
34 mins	4.029	-0.867	0.2170	NS
36 mins	2.560	-0.077	0.4720	NS
38 mins	2.493	-0.611	0.2960	NS
40 mins	3.279	1.943	0.0695	NS
**Post-HFS**				
2 mins	2.012	-3.612	**0.0340**	*
4 mins	3.528	-16.101	**<0.01**	**
6 mins	2.042	-1.920	0.0960	NS
8 mins	2.004	-2.121	0.0840	NS
10 mins	2.035	-2.563	0.0610	NS
12 mins	2.017	-3.230	**0.0415**	*
14 mins	2.120	-1.486	0.1345	NS
16 mins	2.016	-1.739	0.1115	NS
18 mins	2.164	-2.089	0.0810	NS
20 mins	2.512	-1.936	0.0830	NS
22 mins	2.065	-1.354	0.1525	NS
24 mins	2.113	-1.750	0.1075	NS
26 mins	2.203	-1.885	0.0940	NS
28 mins	2.480	-5.535	**<0.01**	**
30 mins	2.044	-2.523	0.0625	NS
32 mins	2.165	-1.869	0.0965	NS
34 mins	2.050	-0.888	0.2330	NS
36 mins	2.034	-1.717	0.1130	NS
38 mins	2.138	-0.306	0.3935	NS
40 mins	2.019	-0.300	0.3960	NS
42 mins	2.005	-0.530	0.3245	NS
44 mins	2.020	-0.869	0.2380	NS

46 mins	2.020	-0.533	0.3235	NS
48 mins	2.020	-0.424	0.3560	NS
50 mins	2.012	-0.328	0.3870	NS
52 mins	2.002	-0.960	0.2190	NS
54 mins	2.150	-0.752	0.2630	NS
56 mins	2.015	-0.282	0.4020	NS
58 mins	2.443	-1.302	0.1510	NS
60 mins	2.330	-3.172	**0.0355**	*

**Independent samples t-test**	**DF**	**t-value**	**p-value**	**Significance**
**Female+SDO vs Female +Veh (Pre-HFS)**				
2 mins	3.030	-0.117	0.4570	NS
4 mins	3.016	-0.580	0.3010	NS
6 mins	3.432	-0.244	0.4105	NS
8 mins	3.224	-0.522	0.3180	NS
10 mins	3.207	0.164	0.4395	NS
12 mins	4.511	0.735	0.2490	NS
14 mins	3.109	0.077	0.4720	NS
16 mins	3.101	-0.428	0.3485	NS
18 mins	3.179	-0.428	0.3480	NS
20 mins	3.062	0.827	0.2340	NS
22 mins	3.406	-0.234	0.4140	NS
24 mins	3.378	1.049	0.1815	NS
26 mins	4.879	-0.032	0.4880	NS
28 mins	3.723	-1.031	0.1825	NS
30 mins	3.902	1.185	0.1515	NS
32 mins	4.767	0.631	0.2785	NS
34 mins	5.686	0.260	0.4020	NS
36 mins	3.434	-1.134	0.1650	NS
38 mins	3.924	1.193	0.1500	NS
40 mins	5.509	2.221	**0.0360**	*

**Post-HFS**				
2 mins	3.014	1.167	0.1635	NS
4 mins	3.812	-2.177	**0.0490**	*
6 mins	3.408	0.996	0.1925	NS
8 mins	3.013	1.506	0.1145	NS
10 mins	3.046	-0.066	0.4755	NS
12 mins	3.020	-0.188	0.4315	NS
14 mins	3.237	-0.379	0.3640	NS
16 mins	3.024	-0.302	0.3910	NS
18 mins	3.099	-0.375	0.3660	NS
20 mins	3.238	0.557	0.3070	NS
22 mins	3.043	0.160	0.4415	NS
24 mins	3.112	1.024	0.1895	NS
26 mins	3.174	0.225	0.4180	NS
28 mins	3.303	-0.326	0.3820	NS
30 mins	3.658	-3.857	**0.0105**	*
32 mins	4.148	-0.896	0.2095	NS
34 mins	4.037	-3.525	**0.0120**	*
36 mins	3.289	-2.761	**0.0315**	*
38 mins	4.135	-0.808	0.2315	NS
40 mins	3.507	-1.872	0.0725	NS
42 mins	3.053	-2.912	**0.0305**	*
44 mins	3.328	-3.186	**0.0215**	*
46 mins	3.117	-1.176	0.1610	NS
48 mins	3.162	-0.829	0.2325	NS
50 mins	3.053	-0.412	0.3535	NS
52 mins	3.022	-1.386	0.1295	NS
54 mins	5.179	-2.535	**0.0255**	*
56 mins	3.207	-0.908	0.2135	NS

58 mins	4.476	-2.289	**0.0385**	*
60 mins	3.376	-1.685	0.0900	NS

Independent samples t-test	DF	t-value	p-value	Significance
**Female+Don vs Female +Veh (Pre-HFS)**				
2 mins	2.060	0.575	0.3110	NS
4 mins	2.148	-0.182	0.4355	NS
6 mins	4.775	0.229	0.4140	NS
8 mins	4.750	-0.589	0.2915	NS
10 mins	4.643	0.687	0.2625	NS
12 mins	2.599	-0.388	0.3640	NS
14 mins	2.398	0.837	0.2390	NS
16 mins	3.256	0.260	0.4050	NS
18 mins	4.245	-1.544	0.0970	NS
20 mins	2.584	0.924	0.2165	NS
22 mins	4.736	1.667	0.0800	NS
24 mins	4.547	0.588	0.2925	NS
26 mins	3.779	1.008	0.1865	NS
28 mins	4.594	-0.723	0.2525	NS
30 mins	2.496	-0.335	0.3820	NS
32 mins	3.767	0.516	0.3175	NS
34 mins	3.018	-0.969	0.2020	NS
36 mins	4.986	0.343	0.3730	NS
38 mins	3.256	1.606	0.0995	NS
40 mins	4.809	-0.001	0.4995	NS
**Post-HFS**				
2 mins	2.009	-3.158	**0.0435**	*
4 mins	2.086	-2.463	0.0640	NS

6 mins	2.103	-2.153	0.0790	NS
8 mins	2.006	-2.366	0.0705	NS
10 mins	2.080	-2.714	0.0545	NS
12 mins	2.019	-2.225	0.0775	NS
14 mins	2.242	-2.491	0.0585	NS
16 mins	2.035	-2.810	0.0525	NS
18 mins	2.137	-2.416	0.0645	NS
20 mins	2.579	-2.969	**0.0355**	*
22 mins	2.078	-3.317	**0.0380**	*
24 mins	2.107	-3.129	**0.0415**	*
26 mins	2.336	-3.684	**0.0260**	*
28 mins	2.203	-3.836	**0.0265**	*
30 mins	2.048	-2.743	0.0540	NS
32 mins	2.269	-2.188	0.0725	NS
34 mins	2.103	-2.871	**0.0485**	*
36 mins	2.071	-3.453	**0.0355**	*
38 mins	2.674	-2.361	0.0550	NS
40 mins	2.071	-2.739	0.0535	NS
42 mins	2.014	-3.602	**0.0340**	*
44 mins	2.058	-2.453	0.0650	NS
46 mins	2.053	-2.932	**0.0480**	*
48 mins	2.048	-2.441	0.0660	NS
50 mins	2.020	-2.220	0.0775	NS
52 mins	2.006	-2.940	**0.0490**	*
54 mins	2.250	-2.494	0.0580	NS
56 mins	2.024	-2.231	0.0770	NS
58 mins	2.217	-2.692	0.0515	NS
60 mins	2.088	-2.391	0.0670	NS

*p <0.05, ** p <0.01 compared to Female+Veh group.

Independent samples t-test	DF	t-value	p-value	Significance
**Male+SL vs Male +Veh (Pre-HFS)**				
2 mins	11.487	0.000	0.5000	NS
4 mins	12.628	1.139	0.1380	NS
6 mins	11.765	1.869	0.0435	*
8 mins	12.628	1.407	0.0915	NS
10 mins	11.487	0.537	0.3010	NS
12 mins	11.487	0.201	0.4220	NS
14 mins	12.736	-0.613	0.2750	NS
16 mins	12.238	0.256	0.4010	NS
18 mins	11.765	0.879	0.1985	NS
20 mins	11.642	0.786	0.2240	NS
22 mins	11.487	0.671	0.2575	NS
24 mins	12.736	-0.068	0.4735	NS
26 mins	12.914	-0.817	0.2145	NS
28 mins	12.736	-0.816	0.2145	NS
30 mins	11.487	-0.805	0.2185	NS
32 mins	12.736	0.068	0.4735	NS
34 mins	12.914	0.954	0.1790	NS
36 mins	12.736	0.136	0.4470	NS
38 mins	11.487	-0.671	0.2575	NS
40 mins	10.817	-0.629	0.2710	NS
**Post-HFS**				
2 mins	12.483	1.300	0.1085	NS
4 mins	12.302	0.360	0.3625	NS
6 mins	12.141	-0.410	0.3445	NS
8 mins	12.862	-0.469	0.3235	NS

10 mins	12.923	-0.530	0.3025	NS
12 mins	12.923	-0.530	0.3025	NS
14 mins	12.923	-0.530	0.3025	NS
16 mins	12.979	-0.498	0.3135	NS
18 mins	12.741	-0.469	0.3235	NS
20 mins	12.484	-0.280	0.3920	NS
22 mins	12.141	-0.082	0.4680	NS
24 mins	12.484	-0.440	0.3335	NS
26 mins	12.741	-0.781	0.2245	NS
28 mins	12.741	-0.781	0.2245	NS
30 mins	12.741	-0.781	0.2245	NS
32 mins	12.741	-1.210	0.1240	NS
34 mins	12.741	-1.640	0.0625	NS
36 mins	12.741	-1.640	0.0625	NS
38 mins	12.741	-1.640	0.0625	NS
40 mins	12.741	-1.640	0.0625	NS
42 mins	12.741	-1.640	0.0625	NS
44 mins	12.741	-1.562	0.0715	NS
46 mins	12.741	-1.484	0.0810	NS
48 mins	12.741	-1.718	0.0550	NS
50 mins	12.741	-1.952	0.0365	*
52 mins	12.338	-2.279	0.0205	*
54 mins	11.871	-2.565	0.0125	*
56 mins	12.862	-2.497	0.0135	*
58 mins	12.923	-2.416	0.0155	*
60 mins	12.862	-2.433	0.0150	*

**Independent samples t-test**	**DF**	**t-value**	**p-value**	**Significance**

Male+SDO vs Male +Veh (Pre-HFS)				
2 mins	11.487	0.000	0.5000	NS
4 mins	12.000	0.776	0.2265	NS
6 mins	10.210	1.302	0.1110	NS
8 mins	11.158	1.255	0.1175	NS
10 mins	9.575	0.876	0.2015	NS
12 mins	10.606	0.630	0.2710	NS
14 mins	11.487	0.403	0.3475	NS
16 mins	12.903	0.237	0.4080	NS
18 mins	12.989	0.000	0.5000	NS
20 mins	11.444	0.735	0.2385	NS
22 mins	12.546	0.299	0.3850	NS
24 mins	12.903	0.632	0.2695	NS
26 mins	12.989	1.001	0.1675	NS
28 mins	12.986	-0.148	0.4420	NS
30 mins	12.983	-1.068	0.1525	NS
32 mins	12.935	-1.413	0.0905	NS
34 mins	12.546	-1.794	0.0485	NS
36 mins	12.986	-1.187	0.1285	NS
38 mins	12.267	-0.582	0.2855	NS
40 mins	12.983	-0.935	0.1835	NS
**Post-HFS**				
2 mins	11.914	0.278	0.3925	NS
4 mins	12.333	-0.317	0.3785	NS
6 mins	12.656	-0.881	0.1975	NS
8 mins	12.680	-1.074	0.1515	NS
10 mins	12.719	-1.243	0.1180	NS
12 mins	12.719	-1.243	0.1180	NS

14 mins	12.719	-1.243	0.1180	NS
16 mins	12.719	-1.476	0.0820	NS
18 mins	12.719	-1.709	0.0560	NS
20 mins	12.437	-1.633	0.0635	NS
22 mins	12.093	-1.549	0.0735	NS
24 mins	12.437	-1.952	0.0370	*
26 mins	12.719	-2.330	0.0185	*
28 mins	12.719	-2.214	0.0230	*
30 mins	12.719	-2.097	0.0285	*
32 mins	12.719	-1.942	0.0375	*
34 mins	12.719	-1.787	0.0490	*
36 mins	12.515	-2.144	0.0260	*
38 mins	12.267	-2.473	0.0145	*
40 mins	12.267	-2.437	0.0155	*
42 mins	12.267	-2.400	0.0165	*
44 mins	12.267	-1.964	0.0365	*
46 mins	12.267	-1.527	0.0760	NS
48 mins	12.267	-1.637	0.0635	NS
50 mins	12.267	-1.746	0.0530	NS
52 mins	12.267	-2.364	0.0175	*
54 mins	11.871	-2.565	0.0055	**
56 mins	12.987	-2.832	0.0070	**
58 mins	12.713	-2.681	0.0095	**
60 mins	12.987	-2.666	0.0095	**

**Independent samples t-test**	**DF**	**t-value**	**p-value**	**Significance**
**Male+Don vs Male +Veh (Pre-HFS)**				
2 mins	12.000	0.237	0.4085	NS

4 mins	11.926	0.799	0.2200	NS
6 mins	11.614	1.178	0.1310	NS
8 mins	11.991	1.104	0.1455	NS
10 mins	9.575	0.876	0.2015	NS
12 mins	10.606	0.630	0.2710	NS
14 mins	12.000	0.403	0.3475	NS
16 mins	11.644	0.071	0.4720	NS
18 mins	11.659	0.482	0.3195	NS
20 mins	11.850	1.055	0.1560	NS
22 mins	11.632	0.643	0.2665	NS
24 mins	11.644	0.357	0.3635	NS
26 mins	11.659	0.000	0.5000	NS
28 mins	11.644	-0.643	0.2665	NS
30 mins	11.632	-1.157	0.1355	NS
32 mins	11.937	-1.023	0.1630	NS
34 mins	11.974	-0.870	0.2005	NS
36 mins	11.150	-1.271	0.1150	NS
38 mins	9.405	-1.581	0.0735	NS
40 mins	11.188	-1.575	0.0715	NS
**Post-HFS**				
2 mins	10.100	-0.885	0.1985	NS
4 mins	10.570	-1.297	0.1110	NS
6 mins	10.990	-1.688	0.0595	NS
8 mins	11.026	-1.703	0.0585	NS
10 mins	11.084	-1.713	0.0570	NS
12 mins	11.084	-1.825	0.0475	*
14 mins	11.084	-1.937	0.0395	*
16 mins	11.084	-1.601	0.0685	NS
18 mins	11.084	-1.266	0.1155	NS

20 mins	10.698	-1.449	0.0880	NS
22 mins	10.294	-1.635	0.0660	NS
24 mins	10.698	-1.563	0.0735	NS
26 mins	12.719	-2.330	0.0185	*
28 mins	11.084	-1.601	0.0685	NS
30 mins	11.084	-1.713	0.0570	NS
32 mins	11.644	-1.713	0.0570	NS
34 mins	11.084	-1.266	0.1155	NS
36 mins	10.796	-1.981	0.0370	*
38 mins	10.491	-2.225	0.0245	*
40 mins	10.491	-2.260	0.0230	*
42 mins	10.491	-2.294	0.0220	*
44 mins	10.491	-2.329	0.0205	*
46 mins	10.491	-2.364	0.0195	*
48 mins	10.491	-1.773	0.0525	NS
50 mins	10.491	-1.182	0.1315	NS
52 mins	10.491	-1.877	0.0445	*
54 mins	10.491	-2.573	0.0135	*
56 mins	11.647	-2.823	0.0080	**
58 mins	12.000	-3.010	0.0055	**
60 mins	11.647	-2.566	0.0125	*

*p <0.05, ** p <0.01 compared to Male+Veh group.
